# Benzodiazepine Discontinuation and Mortality Among Patients Receiving Long-Term Benzodiazepine Therapy

**DOI:** 10.1001/jamanetworkopen.2023.48557

**Published:** 2023-12-20

**Authors:** Donovan T. Maust, Kierstdea Petzold, Julie Strominger, H. Myra Kim, Amy S. B. Bohnert

**Affiliations:** 1 Department of Psychiatry, University of Michigan, Ann Arbor; 2 Center for Clinical Management Research, VA Ann Arbor Healthcare System, Ann Arbor, Michigan; 3 Institute for Healthcare Policy and Innovation, University of Michigan, Ann Arbor; 4 Center for Statistical Consultation and Research, University of Michigan, Ann Arbor; 5 Department of Anesthesiology, University of Michigan, Ann Arbor

## Abstract

**Importance:**

There is interest in reducing long-term benzodiazepine prescribing given harms associated with use, but the cumulative risks or benefits of discontinuation are unknown.

**Objective:**

To identify the association of benzodiazepine discontinuation with mortality and other adverse events among patients prescribed stable long-term benzodiazepine therapy, stratified by baseline opioid exposure.

**Design, Setting, and Participants:**

This comparative effectiveness study with a trial emulation approach included data from a US commercial insurance database between January 1, 2013, and December 31, 2017. Eligible participants were adults with stable long-term benzodiazepine prescription treatment. Data were analyzed between December 2022 and November 2023.

**Exposure:**

Benzodiazepine discontinuation, defined as no benzodiazepine prescription coverage for 31 consecutive days identified during a 6-month grace period after baseline.

**Main Outcome and Measures:**

Mortality during 12 months of follow-up; secondary outcomes included nonfatal overdose, suicide attempt or self-inflicted injury, suicidal ideation, and emergency department use, identified in medical claims. Inverse probability weighting was used to adjust for baseline confounders that potentially affected treatment assignment and censoring due to death or disenrollment. Primary analysis used an intention-to-treat approach; a secondary per-protocol analysis estimated associations after accounting for nonadherence. Analyses were stratified by opioid use.

**Results:**

The study included 213 011 (136 609 female [64.1%]; mean [SD] age, 62.2 [14.9] years; 2953 Asian [1.4%], 18 926 Black [8.9%], 22 734 Hispanic [10.7%], and 168 398 White [60.2%]) and 140 565 (91 811 female [65.3%]; mean [SD] age, 61.1 [13.2] years; 1319 Asian [0.9%], 15 945 Black [11.3%], 11 989 Hispanic [8.5%], and 111 312 White [79.2%]) patients with stable long-term benzodiazepine use without and with opioid exposure, respectively. Among the nonopioid exposed, the adjusted cumulative incidence of death after 1 year was 5.5% (95% CI, 5.4%-5.8%) for discontinuers, an absolute risk difference of 2.1 percentage points (95% CI, 1.9-2.3 percentage points) higher than for nondiscontinuers. The mortality risk was 1.6 (95% CI, 1.6-1.7) times that of nondiscontinuers. Among those with opioid exposure, the adjusted cumulative incidence of death was 6.3% (95% CI, 6.0%-6.6%) for discontinuers, an absolute risk difference of 2.4 percentage points (95% CI, 2.2-2.7 percentage points) higher than for nondiscontinuers and a mortality risk 1.6 (95% CI, 1.5-1.7) times that of nondiscontinuers. Cumulative incidence of secondary outcomes was also higher among discontinuers.

**Conclusions and Relevance:**

This study identifies small absolute increases in risk of harms among patients with stable long-term prescription benzodiazepine treatment who appear to discontinue relative to continuing treatment, including those with and without recent prescription opioid exposure. Policy broadly promoting benzodiazepine discontinuation may have unintended risks.

## Key Points

**Question:**

Given the association of benzodiazepine receipt with patient harms, does prescription discontinuation reduce risk of death and other harms in patients receiving stable long-term benzodiazepine treatment?

**Findings:**

In this comparative effectiveness study among 353 576 patients receiving stable long-term treatment with benzodiazepines, discontinuation was associated with small absolute increases in mortality and other potential harms, including nonfatal overdose, suicide attempt, suicidal ideation, and emergency department visits.

**Meaning:**

These results suggest benzodiazepine discontinuation among patients prescribed for stable long-term treatment may be associated with unanticipated harms, and that efforts to promote discontinuation should carefully consider the potential risks of discontinuation relative to continuation.

## Teaser

This comparative effectiveness study of US adults receiving long-term treatment with benzodiazepines examines the association of treatment discontinuation with mortality and adverse events related to addiction and mental health.

## Introduction

Over 10% of US adults reported past-year prescription benzodiazepine use from 2015-2016,^1^ with the prevalence and total volume of prescribing having grown over the prior 2 decades.^2^ After opioids, benzodiazepine are the medication most involved in prescription overdose (OD) deaths^3^; by 2020, the US had the highest ever number of benzodiazepine-involved OD deaths.^4^ The growth in use and associated harms may account for increased attention from the US Food and Drug Administration (FDA), with a 2016 warning related to coprescribing with opioids^5^; a 2020 class-wide boxed warning regarding “the risks of abuse, misuse, addiction, physical dependence, and withdrawal reactions”^6^; and an Evidence-Based Clinical Practice Guideline for the Safe Tapering of Benzodiazepines in progress.^7^

The FDA guideline development announcement is focused on reducing long-term benzodiazepine use in a manner that is consistent with expert opinion and professional guidelines, which suggest that short-term or intermittent benzodiazepine use has a more favorable risk-benefit profile.^8,9,10^ While extensive evidence characterizes the risks associated with benzodiazepine use,^3,11,12,13,14^ no studies have focused explicitly on discontinuation risks. Conventional wisdom might suggest benzodiazepine discontinuation would reduce risk of adverse events, yet this has not been the case with long-term prescription opioid discontinuation.^15,16,17,18,19^ In fact, cessation of treatment may be particularly fraught for benzodiazepine: psychologically, because many patients find the prospect of discontinuation distressing,^20,21^ and physiologically, because of physical dependence and the potential for withdrawal, including seizures.^8,22,23^

To inform benzodiazepine policy and guidelines, we sought to determine the cumulative risks or benefits associated with benzodiazepine discontinuation. We did so using an emulated target trial approach in national claims data^24^ by identifying a population of patients receiving stable long-term benzodiazepine treatment and estimating the association of discontinuation with all-cause mortality and other potential associated harms. Given the increased mortality associated with coprescribed opioids and benzodiazepine, we stratified analysis by opioid use to inform decision-making for patients coprescribed opioids. We hypothesized that prescription benzodiazepine discontinuation would be associated with reduced risk of mortality and other adverse events.

## Methods

### Study Design and Data Source

We used a commercial US health claims database (Optum) for 2013 through 2019 to conduct an analysis examining the association between benzodiazepine discontinuation and mortality, nonfatal overdose, suicide attempt or self-inflicted injury, suicidal ideation, and emergency department use among long-term benzodiazepine users. The deidentified health claims data represent a large sample from across the US, including commercially insured (ie, working age) and Medicare Advantage (ie, older adult) beneficiaries. The study was approved by the Michigan Medicine institutional review board for retrospective analysis of deidentified secondary data with a waiver of informed consent and followed the International Society for Pharmacoeconomics and Outcomes Research (ISPOR) reporting guidelines.

### Eligibility Criteria

We established a cohort of adults with stable long-term benzodiazepine consumption by identifying all benzodiazepine prescriptions filled between January 1, 2013, and December 31, 2019, using pharmacy claims (hereafter, *index fills*) (eFigure in Supplement 1). We restricted to index fills where the patient was alive and had continuous insurance coverage for the next 365 days, which was the baseline period. We excluded patients with fills for liquid benzodiazepine given difficulties computing average daily dose in lorazepam-equivalent doses for liquid benzodiazepine prescriptions.^25^

To limit the cohort to long-term benzodiazepine use, we used prescription fill dates and days dispensed to restrict index fills to those with benzodiazepine coverage for 90% of days or more during the baseline year and no gaps in benzodiazepine coverage greater than 30 consecutive days. We required long-term use to reflect a relatively consistent, continuous level of benzodiazepine exposure so that discontinuation (described in “Treatment Strategies and Assignment”) represented a marked change in treatment. Finally, to limit the cohort to stable long-term therapy, we further limited to those with index fills followed by a year-long baseline with monthly average daily doses within 30% of the baseline grand mean using lorazepam-equivalent doses.^25^ We removed index fills where the patient was younger than 18 years, received hospice care during baseline, or had cancer (except nonmelanoma skin cancer), a seizure disorder, or a nonfatal overdose during baseline (eTable 1 in Supplement 1).

We removed patients missing key variables or a baseline average daily benzodiazepine dose greater than the 99th percentile (15.8 lorazepam-equivalent mg/d). These doses may have resulted from data error, but if accurate, patients receiving such high-dose therapy would require an alternative discontinuation approach. We emulated the trial separately for those recently exposed to opioids (ie, received 1 or more opioid fill during the last 30 days of baseline) to examine the effect size associated with discontinuation by baseline opioid use status. Finally, within each potentially eligible target population receiving stable long-term benzodiazepine therapy, a given patient could have multiple index fills; we randomly selected 1 index fill per patient.^24^

### Outcome and Follow-Up

**Figure 1.**
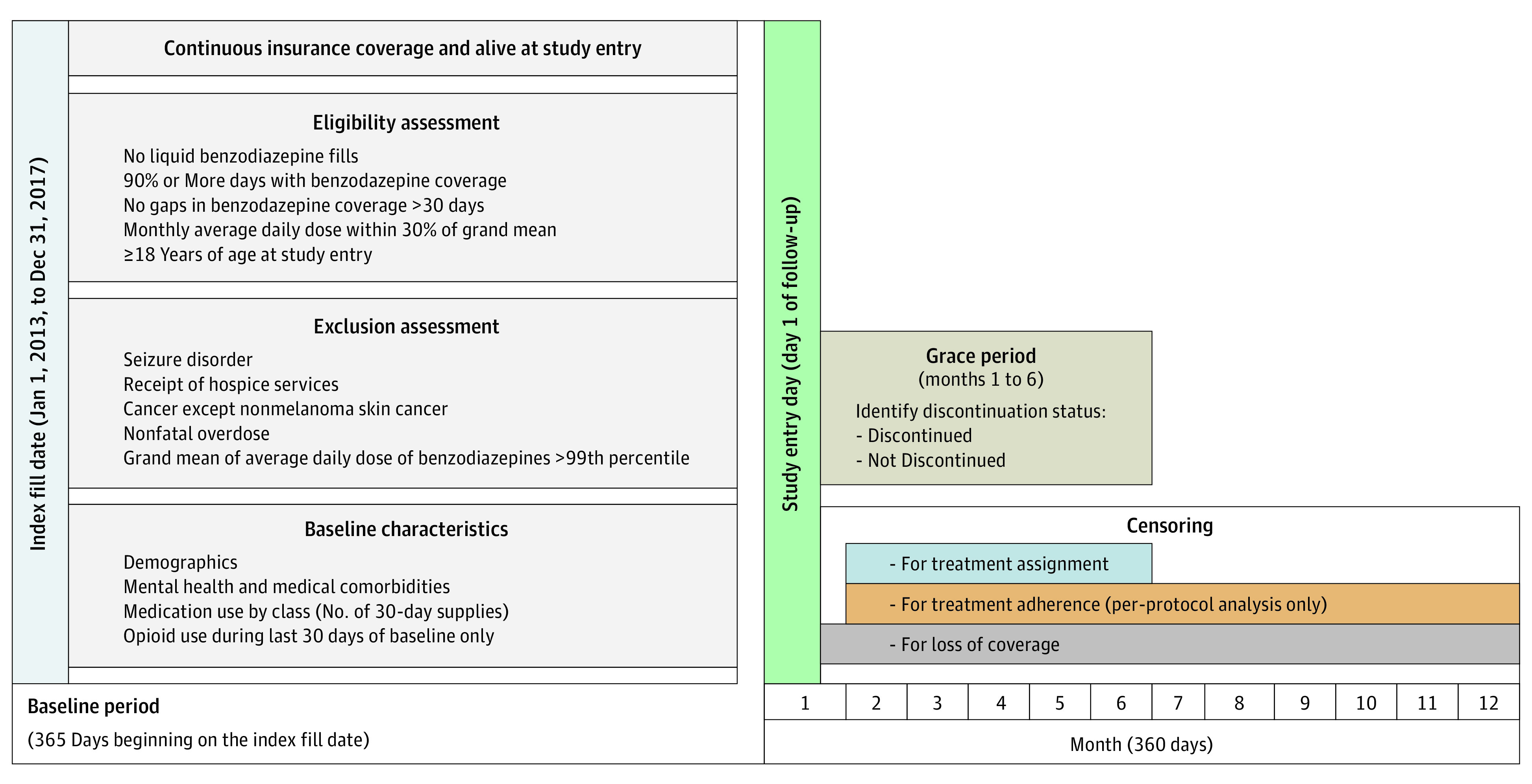
Study Design for Target Trial Emulation of Discontinuation From Long-Term Stable Benzodiazepine Prescriptions

The primary outcome was all-cause mortality. Follow-up started immediately after the end of baseline year, was 360 days long (allowing twelve 30-day months), and ended the month during which the outcome was observed or insurance disenrollment occurred, whichever came first (Figure 1). Because only month and year of death are available in the data (for deidentification purposes), we randomly assigned day of death within the month of death, assuming a uniform distribution across days.^26^ Although we used discrete time survival methods (discretized to month), we assigned a date of death to ascertain if death occurred before or after benzodiazepine discontinuation.

### Treatment Strategies and Assignment

An outlined protocol of the target trial we sought to emulate is presented in Table 1. We compared 2 treatment strategies: (1) discontinuation from stable long-term benzodiazepine therapy or (2) continued benzodiazepine prescribing. Treatment strategy was assessed during a 180-day grace period following baseline, providing a time frame from randomization to potential discontinuation consistent with, or longer than, benzodiazepine discontinuation randomized trials.^27,28,29,30,31^ Patients were considered discontinued beginning on the 31st consecutive day with no benzodiazepine coverage.

**Table 1.** Protocol of the Target Trial and Emulation Examining Discontinuation of Benzodiazepines Among Patients Prescribed Stable Long-term Benzodiazepine Therapy

Characteristic	Specifying target trial	Target trial emulation
Eligibility criteria		
Inclusion	Continuous insurance coverage during a 1-y baseline and alive at start of follow-up; benzodiazepine prescription supply covering ≥90% of days (excluding liquid fills) during 1-y baseline; no gaps in benzodiazepine supply of >30 d during 1-y baseline; stable benzodiazepine prescription supply, defined as each monthly average daily lorazepam-equivalent dose within 30% of grand mean during 1-y baseline; age ≥18 y at start of follow-up	Same as target trial
Exclusion	Seizure disorder, hospice use, nonmelanoma cancer, overdose during baseline	Same as target trial
Treatment strategies	Discontinue benzodiazepine treatment within 6 mo (intervention); continue benzodiazepine treatment (control)	Discontinue benzodiazepine prescription fills within 6 mo (defined as >30 consecutive d with no benzodiazepine coverage) (intervention); continue benzodiazepine prescription fills (control)
Treatment assignment	Randomly assign eligible patients to treatment groups; patients are aware of the treatment they are assigned to. Stratify by opioid use	Treatment assignment emulated using the clone-censor-weighting approach with 6-mo grace period. Each patient is cloned and starts within both treatment groups; clones are censored during the grace period when they are no longer adherent with corresponding treatment (ie, those who discontinue have their do-not-discontinue clone censored when they discontinue [day 31 with no benzodiazepine coverage]). Potential selection bias introduced by censoring adjusted using inverse probability of censoring weights. Stratify by opioid use.
Follow-up		
Start of follow-up	Eligibility criteria met	Eligibility criteria met
End of follow-up	Outcome observed, censored, or end of follow-up (360 d)	Outcome observed, censored, or end of follow-up (360 d)
Censoring	Lost to follow-up, death (if not outcome), lack of adherence (in per-protocol)	Treatment no longer compatible with the treatment strategy for the group, death (if not outcome), loss of coverage
Outcomes		
Primary	Death	Death
Secondary	Nonfatal overdose; suicide attempt or self-inflicted injury; suicidal ideation; emergency department visits	Measured using claims data and diagnosis codes: nonfatal overdose (identified at emergency department visits or hospitalization); suicide attempt or self-inflicted injury; suicidal ideation; emergency department visits
Causal contrast	Intention-to-treat; per-protocol	Observational analogs of intention-to-treat and per-protocol
Analysis plan^a^		
Intention-to-treat	Compare 360-d risk of mortality between patients assigned to each treatment using pooled logistic regression	Weighted pooled logistic regression model to examine the association between treatment and mortality; final weight based on 2 inverse probability weights (treatment assignment and loss-to-follow-up)
Per-protocol	Same as the intention-to-treat except additionally adjusted for nonadherence to the assigned treatment by computing and applying inverse probability weights to account for the association between baseline covariates and nonadherence. Standardized cumulative incidence curves created	Weighted pooled logistic regression model to examine the association between treatment and mortality; final weight based on 3 inverse probability weights (treatment assignment, loss-to-follow-up, and non-adherence)
Baseline covariates	Age, sex, race, census region, average benzodiazepine use in lorazepam-equivalent units, year at start of follow-up, medication use during baseline, modified Elixhauser score, mental health and clinical comorbidities	Age, sex, race, census region, average benzodiazepine use in lorazepam-equivalent units, year at start of follow-up, medication use during baseline, modified Elixhauser score, mental health and clinical comorbidities

^a^ Analyses stratified by baseline opioid use.

We used the clone-censor-weight approach to overcome immortal time and other biases potentially present when time anchors overlap, such as during the treatment assignment period and follow-up here.^24,32^ We created 2 clones for each patient, where 1 was assigned to the discontinued benzodiazepine group and the other to the continued benzodiazepine group. We censored clones when their observed benzodiazepine fills were no longer consistent with the assigned treatment strategy. For patients who did not discontinue benzodiazepine during the grace period, their discontinued benzodiazepine clone was censored at the end of the grace period, and 1 clone per patient remained at the end of the grace period. Under the clone-censor-weight approach, events that occur during the grace period but prior to either clone being censored are attributed to both groups.

### Estimates of Effect Sizes

We estimated both intention-to-treat and per-protocol effect sizes of discontinuation from stable long-term benzodiazepine prescribing on our outcomes of interest. When estimating the intent-to-treat effect sizes, once a patient was assigned to a single treatment group, they were assigned to the group for the entirety of follow-up regardless of treatment adherence status. In contrast, when estimating the per-protocol effect sizes, after each patient was assigned to a single treatment group, we censored when they were no longer adherent to the assigned treatment.

### Potential Confounders

We adjusted for the following potential confounding factors, measured during baseline: age; sex; race and ethnicity; census region; year at start of follow-up; average daily lorazepam-equivalent benzodiazepine dose; anxiety; depression; long-term noncancer pain; insomnia; bipolar disorder; other psychotic disorders; substance use disorders (alcohol, opioid, cocaine and stimulants, sedatives, cannabis, and other substances); a modified Elixhauser comorbidity score (excluding mental health conditions captured separately); and 30-day supplies of antidepressants, antipsychotics, antiepileptics, and nonbenzodiazepine benzodiazepine receptor agonists (eTables 2 and 3 in Supplement 1). Race and ethnicity is provided within the claims database (Asian, Black, Hispanic, White, unknown, or missing) and was included to capture unmeasured social factors, including the experience of racism.

### Statistical Analysis

To allow for potential differences in the association between discontinuation from stable long-term benzodiazepine use and mortality by opioid use, all analyses were stratified by opioid use. We examined the association between discontinuation from stable long-term benzodiazepine prescriptions and mortality using discrete time survival analysis and fitting a weighted pooled logistic regression model with treatment group, month (included as linear, quadratic, and cubic terms), and interaction terms between treatment group and month. We estimated 3 sets of time-varying weights: (1) a treatment assignment weight to adjust for informative censoring of clones during the grace period, (2) a loss-to-follow-up weight to adjust for possible selection bias due to insurance disenrollment, and (3) a treatment adherence weight (only for per-protocol analysis) to adjust for confounding related to nonadherence. Each weight was estimated separately as the inverse probability of remaining uncensored at each month for each patient given potential confounders, and the probability of remaining uncensored up to the month was calculated as the cumulative product of the monthly weights (eMethods in Supplement 1). We truncated the final weight at the 99th percentile.

After fitting the weighted logistic regression model, we computed and plotted the standardized cumulative incidence by treatment group. Additionally, we calculated the risk difference and risk ratio to compare benzodiazepine discontinuation with continuation 12 months after baseline. We estimated percentile-based 95% CIs using bootstrapping with 100 samples. Significance was set at α = .05; tests were 2-sided. Analysis was conducted using SAS Enterprise Guide version 8.1 (SAS Institute).

We repeated the analysis for the following secondary outcomes: nonfatal overdose; suicide attempt or self-inflicted injury; suicidal ideation; and any emergency department use (eTable 4 in Supplement 1). For secondary outcomes, the loss-to-follow-up weight to account for disenrollment also accounted for death. Given potential age differences in risk, we conducted a secondary analysis of the primary outcome in age subgroups. Lastly, we performed a sensitivity analysis of the primary outcome by defining benzodiazepine discontinuation as 61 consecutive days without benzodiazepine coverage.

## Results

There were 221 512 patients without recent opioid exposure and 145 954 patients with recent opioid exposure with stable, long-term benzodiazepine use that met our inclusion and exclusion criteria (eFigure in Supplement 1). Of those, 8501 (3.8%) and 5389 (3.7%), respectively, were excluded due to missing information on key covariates (eg, race and ethnicity), resulting in 213 011 patients without recent opioid exposure (136 609 female [64.1%]; mean [SD] age, 62.2 [14.9] years; 2953 Asian [1.4%], 18 926 Black [8.9%], 22 734 Hispanic [10.7%], and 168 398 White [60.2%]) and 140 565 patients with recent opioid exposure (91 811 female [65.3%]; mean [SD] age, 61.1 [13.2] years; 1319 Asian [0.9%], 15 945 Black [11.3%], 11 989 Hispanic [8.5%], and 111 312 White [79.2%]).

### Intention-to-Treat Results

Characteristics of stable long-term benzodiazepine users in the intention-to-treat analysis are presented in Table 2. Among patients without recent opioid exposure, treatment groups were reasonably balanced prior to any adjustment, except noncancer pain was slightly higher among discontinuers vs nondiscontinuers (27 902 of 38 010 [73.4%] vs 100 577 of 140 924 [71.4%]), while anxiety was lower (18 805 of 38 010 [49.5%] vs 73 168 of 140 924 [51.9%]). Among patients with recent opioid exposure, insomnia was more common among discontinuers (4643 of 38 010 [20.8%] vs 17 244 of 140 924 [17.8%]). Nondiscontinuers filled a slightly higher volume of prescription medications during baseline (eg, mean [SD] 30-day supplies of antidepressants among patients without opioid exposure: 8.09 [8.87] vs 7.79 [8.57]). After applying the clone-censor-weight approach, these imbalances were largely mitigated (eTables 5 and 6 in Supplement 1).

**Table 2.** Characteristics of Patients Prescribed Stable Long-Term Benzodiazepine Therapy Stratified by Opioid Exposure at the End of the Grace Period for the Intention-to-Treat Analysis, Overall and by Benzodiazepine Discontinuation Status^a^

Characteristic	Patients, No. (%)					
	Without opioid exposure^b^			With opioid exposure^b^		
	Overall	Discontinued	Not discontinued	Overall	Discontinued	Not discontinued
No. (unweighted)	178 934 (100)	38 010 (21.2)	140 924 (78.8)	118 919 (100)	22 287 (18.7)	96 632 (81.3)
Age, y						
18-44	20 808 (11.6)	4740 (12.5)	16 068 (11.4)	11 643 (9.8)	2256 (10.1)	9387 (9.7)
45-64	67 303 (37.6)	13 437 (35.4)	53 866 (38.2)	56 562 (47.6)	10 096 (45.3)	46 466 (48.1)
≥65	90 823 (50.8)	19 833 (52.2)	70 990 (50.4)	50 714 (42.6)	9935 (44.6)	40 779 (42.2)
Sex						
Female	115 539 (64.6)	24 360 (64.1)	91 179 (64.7)	78 001 (65.6)	14 433 (64.8)	63 568 (65.8)
Male	63 395 (35.4)	13 650 (35.9)	49 745 (35.3)	40 918 (34.4)	7854 (35.2)	33 064 (34.2)
Race and ethnicity						
Asian	2506 (1.4)	601 (1.6)	1905 (1.4)	1100 (0.9)	223 (1.0)	877 (0.9)
Black	16 117 (9.0)	3361 (8.8)	12 756 (9.1)	13 638 (11.5)	2671 (12.0)	10 967 (11.3)
Hispanic	19 589 (10.9)	4317 (11.4)	15 272 (10.8)	10 389 (8.7)	2067 (9.3)	8322 (8.6)
White	140 722 (78.6)	29 731 (78.2)	110 991 (78.8)	93 792 (78.9)	17 326 (77.7)	76 466 (79.1)
Region						
Midwest	36 774 (20.6)	7277 (19.1)	29 497 (20.9)	22 708 (19.1)	3834 (17.2)	18 874 (19.5)
Northeast	17 399 (9.7)	3277 (8.6)	14 122 (10.0)	8550 (7.2)	1346 (6.0)	7204 (7.5)
South	91 606 (51.2)	19 240 (50.6)	72 366 (51.4)	65 751 (55.3)	12 410 (55.7)	53 341 (55.2)
West	33 155 (18.5)	8216 (21.6)	24 939 (17.7)	21 910 (18.4)	4697 (21.1)	17 213 (17.8)
Anxiety	91 973 (51.4)	18 805 (49.5)	73 168 (51.9)	67 578 (56.8)	12 230 (54.9)	55 348 (57.3)
Depression	50 304 (28.1)	10 623 (27.9)	39 681 (28.2)	37 339 (31.4)	7052 (31.6)	30 287 (31.3)
Chronic pain	128 479 (71.8)	27 902 (73.4)	100 577 (71.4)	107 289 (90.2)	20 533 (92.1)	86 756 (89.8)
Insomnia	29 841 (16.7)	6985 (18.4)	22 856 (16.2)	21 887 (18.4)	4643 (20.8)	17 244 (17.8)
Bipolar	14 834 (8.3)	2973 (7.8)	11 861 (8.4)	10 556 (8.9)	1849 (8.3)	8707 (9.0)
Other psychotic disorders	6968 (3.9)	1615 (4.2)	5353 (3.8)	3968 (3.3)	775 (3.5)	3193 (3.3)
Substance use disorders						
Alcohol	4554 (2.5)	1171 (3.1)	3383 (2.4)	3416 (2.9)	711 (3.2)	2705 (2.8)
Opioid	4810 (2.7)	1432 (3.8)	3378 (2.4)	10 163 (8.5)	2316 (10.4)	7847 (8.1)
Stimulant	806 (0.5)	265 (0.7)	541 (0.4)	770 (0.6)	194 (0.9)	576 (0.6)
Sedative	4602 (2.6)	1060 (2.8)	3542 (2.5)	2941 (2.5)	617 (2.8)	2324 (2.4)
Cannabis	1143 (0.6)	325 (0.9)	818 (0.6)	1008 (0.8)	228 (1.0)	780 (0.8)
Other substances	2401 (1.3)	712 (1.9)	1689 (1.2)	3531 (3.0)	832 (3.7)	2699 (2.8)
Modified Elixhauser score, mean (SD)^c^	2.67 (2.41)	2.79 (2.55)	2.63 (2.37)	3.17 (2.57)	3.36 (2.71)	3.13 (2.53)
Benzodiazepine dispensed during baseline, mean (SD), lorazepam-equivalent mg/d	2.75 (2.48)	2.47 (2.36)	2.83 (2.50)	3.47 (2.87)	3.03 (2.69)	3.57 (2.90)
Total 30-d medication prescription fills during baseline period, mean (SD), No.						
Antidepressants	8.02 (8.81)	7.79 (8.57)	8.09 (8.87)	8.76 (9.21)	8.55 (9.10)	8.80 (9.24)
Antiepileptics	2.72 (5.65)	2.69 (5.54)	2.73 (5.68)	3.90 (6.35)	4.03 (6.35)	3.86 (6.36)
Antipsychotics	1.73 (5.00)	1.56 (4.68)	1.77 (5.08)	1.61 (4.62)	1.43 (4.31)	1.65 (4.69)
Z-drugs	0.86 (2.94)	0.81 (2.81)	0.87 (2.97)	1.19 (3.42)	1.16 (3.31)	1.20 (3.44)
Year (at start of follow-up)						
2014	27 475 (15.4)	6743 (17.7)	20 732 (14.7)	20 211 (17.0)	4001 (18.0)	16 210 (16.8)
2015	27 191 (15.2)	6568 (17.3)	20 623 (14.6)	18 861 (15.9)	3467 (15.6)	15 394 (15.9)
2016	29 595 (16.5)	6772 (17.8)	22 823 (16.2)	20 942 (17.6)	3852 (17.3)	17 090 (17.7)
2017	37 941 (21.2)	7750 (20.4)	30 191 (21.4)	27 907 (23.5)	5004 (22.5)	22 903 (23.7)
2018	56 732 (31.7)	10 177 (26.8)	46 555 (33.0)	30 998 (26.1)	5963 (26.8)	25 035 (25.9)

^a^ Discontinuation is defined as 31 consecutive days without prescription benzodiazepine coverage. Characteristics presented here from the end of the grace period, by which time each patient has a maximum of 1 clone remaining; overall totals differ from the Abstract and flow chart, which include patients who contributed to outcomes but were censored by the end of the grace period.

^b^ Opioid exposure is defined as the presence of at least one prescription opioid fill during the last 30 days of the baseline period.

^c^ The Elixhauser score is modified to exclude depression, substance abuse, alcohol abuse, and psychosis as these are included as separate covariates.

**Figure 2.**
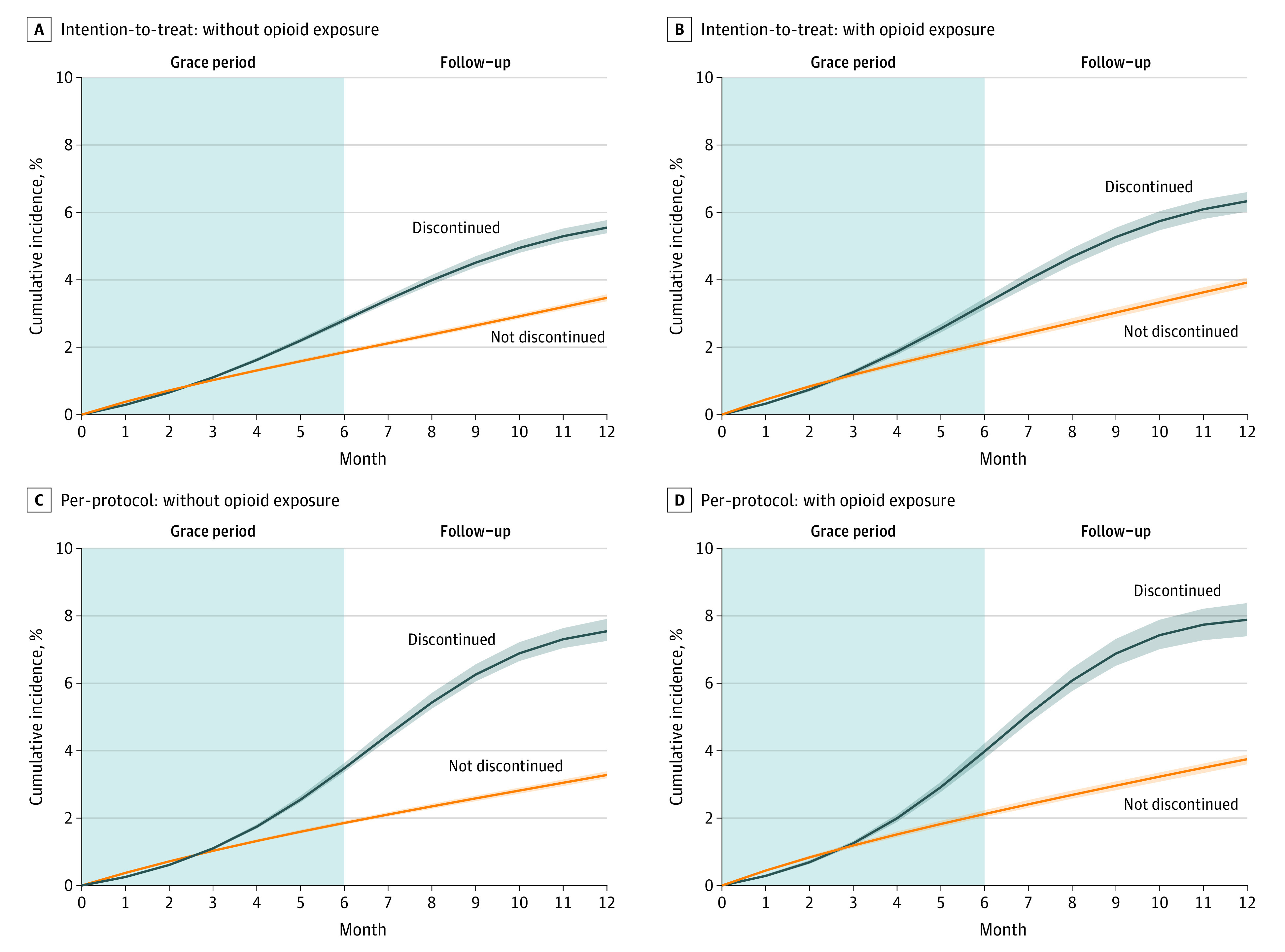
Standardized Cumulative Incidence Curves Examining the Association Between Benzodiazepine Discontinuation and Mortality, Stratified by Opioid Exposure Shaded areas indicate 95% CIs. The grace period is the 6-month period during which a given patient is assigned to a treatment group. Total numbers at risk were 213 011 patients for the analyses of the without opioid exposure group (panels A and C) and 140 565 patients in the opioid exposure group (panels B and D).

Among those without opioid exposure, the adjusted cumulative incidence of death 1 year after the start of follow-up was 5.5% (95% CI, 5.4%-5.8%) for discontinuers and 3.5% (95% CI, 3.4%-3.6%) for nondiscontinuers (Figure 2 and Table 3), corresponding to an absolute risk difference estimate of 2.1 percentage points (95% CI, 1.9-2.3 percentage points) higher among discontinuers. The mortality risk at 12 months for discontinuers without baseline opioid use was 1.6 (95% CI, 1.6-1.7) times that of nondiscontinuers.

**Table 3.** Mortality and Secondary Outcomes by Treatment Strategy Among Patients Prescribed Stable Long-Term Benzodiazepine Therapy, Stratified by Opioid Exposure in the Intention-to-Treat Approach

Outcome	Adjusted cumulative incidence, % (95% CI)^a^		Discontinued (vs not discontinued)	
	Discontinued^b^	Not discontinued	Absolute risk difference, percentage points (95% CI)	Risk ratio (95% CI)
All-cause mortality				
Without opioid exposure^c^	5.5 (5.4-5.8)	3.5 (3.4-3.6)	2.1 (1.9-2.3)	1.6 (1.6-1.7)
With opioid exposure	6.3 (6.0-6.6)	3.9 (3.8-4.1)	2.4 (2.2-2.7)	1.6 (1.5-1.7)
**Secondary outcomes**				
Nonfatal overdose				
Without opioid exposure	1.1 (1.0-1.1)	0.9 (0.9-1.0)	0.1 (0.1-0.2)	1.2 (1.1-1.3)
With opioid exposure	2.2 (2.0-2.3)	1.8 (1.7-1.9)	0.4 (0.2-0.5)	1.2 (1.1-1.3)
Suicide attempt or self-inflicted injury				
Without opioid exposure	0.5 (0.5-0.6)	0.5 (0.4-0.5)	0.1 (0.0-0.1)	1.1 (1.0-1.1)
With opioid exposure	0.8 (0.7-0.9)	0.7 (0.6-0.7)	0.1 (0.0-0.2)	1.2 (1.1-1.4)
Suicidal ideation				
Without opioid exposure	1.0 (0.9-1.1)	0.8 (0.7-0.8)	0.3 (0.2-0.3)	1.4 (1.2-1.5)
With opioid exposure	1.3 (1.1-1.4)	0.9 (0.9-1.0)	0.4 (0.2-0.5)	1.4 (1.2-1.5)
Emergency department use				
Without opioid exposure	42.7 (42.4-43.1)	36.6 (36.4-36.9)	6.1 (5.7-6.5)	1.2 (1.2-1.2)
With opioid exposure	54.3 (53.8-54.8)	45.2 (44.9-45.5)	9.1 (8.5-9.5)	1.2 (1.2-1.2)

^a^ Outcome measure (eg, mortality) computed at the end of follow-up (ie, 360 days).

^b^ Discontinuation is defined as 31 consecutive days without prescription benzodiazepine coverage.

^c^ Opioid exposure is defined as the presence of at least 1 prescription opioid fill during the last 30 days of the baseline period.

Among those with opioid exposure, the adjusted cumulative incidence of death 1 year after start of follow-up was higher, at 6.3% (95% CI, 6.0%-6.6%) for discontinuers and 3.9% (95% CI, 3.8%-4.1%) for nondiscontinuers. The absolute risk difference estimate was 2.4 percentage points (95% CI, 2.2-2.7 percentage points) higher among those who discontinued benzodiazepine vs not, yielding a mortality risk at 12 months for discontinuers 1.6 (95% CI, 1.5-1.7) times that of nondiscontinuers. The relative risk of mortality was similar in analyses with subgroups for both age and opioid use (eTable 7 in Supplement 1).

### Per-Protocol Results

Characteristics of stable long-term benzodiazepine users at the end of the grace period for the per-protocol analysis are presented in eTable 8 in Supplement 1. Among those without recent opioid exposure, balance across treatment groups was similar except discontinuers were older (age 65 years and older: 10 033 of 18 924 [53.0%] vs 70 990 of 140 924 [50.4%]), varied by geography (eg, West: 4603 of 18 924 [24.3%] vs 24 939 of 140 924 [17.7%]), and had more pain (13 952 of 18 924 [73.7%] vs 100 577 of 140 924 [71.4%]) and insomnia (3645 of 18 924 [19.3%] vs 22 856 of 140 924 [16.2%]). Nondiscontinuers filled a slightly higher volume of prescription medications (eg, mean [SD] 30-day supplies of antidepressants among patients without opioid exposure: 8.09 [8.87] vs 7.51 [8.43]). Balance across treatment groups was similar for those with opioid exposure. After applying the clone-censor-weight approach, these small imbalances were mitigated (eTables 9 and 10 in Supplement 1).

Per-protocol results are presented in eTable 11 in Supplement 1. For all outcomes, discontinuers were at higher risk than nondiscontinuers, and the risks were higher than in the intention-to-treat approach. Correspondingly, risk ratios were higher among discontinuers overall, and slightly higher than found in the intention-to-treat approach.

### Secondary Outcomes

There were small increases in the absolute risk differences for nonfatal overdose and suicidal ideation for discontinuers in both those without and with recent opioid exposure (Table 3). For example, the absolute risk difference for those without opioid exposure was 0.1% (95% CI, 0.1%-0.2%) for nonfatal overdose and 0.3% (95% CI, 0.2%-0.3%) for suicidal ideation. There were larger absolute risk differences in emergency department use between groups (eg, without opioid exposure: 6.1%; 95% CI, 5.7%-6.5%). Suicide attempt or self-inflicted injury was not significantly different between groups (eg, without opioid exposure: 0.1%; 95% CI, 0.0%-0.1%). Among discontinuers without opioid exposure, the relative risks for nonfatal overdose, suicidal ideation, and emergency department use increased 1.2 (95% CI, 1.1-1.3), 1.4 (95% CI, 1.2-1.5), and 1.2 (95% CI, 1.2-1.2) times, respectively. For all outcomes, the absolute risk differences for those with opioid exposure (vs without) were slightly higher; risk ratios were similar.

### Sensitivity Analysis Results

Results where we defined discontinuation as 61 consecutive days of no benzodiazepine coverage were consistent with the primary analysis. A cumulative incidence of mortality after 1 year was higher among discontinuers than nondiscontinuers in both the intention-to-treat and per-protocol analyses, and higher among those with recent opioid exposure than those without (eTable 12 in Supplement 1).

## Discussion

In this quality improvement study using target trial emulation of over 350 000 adults prescribed stable long-term benzodiazepine, there was a small absolute increase in mortality among those with their prescription benzodiazepine discontinued over 1 year of follow-up: 2.1 and 2.4 percentage points higher for those without and with recent opioid exposure, respectively. Discontinuation was also associated with absolute increases in nonfatal overdose, suicidal ideation, and emergency department use. While the absolute magnitude of the increased risk for these outcomes was relatively small, the direction of the effect size was not as hypothesized.

Given the increased OD risk and mortality associated with benzodiazepine prescribing,^3^ particularly when coprescribed with opioids,^14,33^ we anticipated that discontinuing benzodiazepine prescriptions would be associated with a lower mortality risk. Indeed, the FDA call to develop benzodiazepine tapering guidelines, part of the US Department of Health and Human Services Overdose Prevention Strategy, is explicitly framed as an effort to minimize risks associated with long-term benzodiazepine use.^7^ However, for every outcome examined in this analysis, discontinuation was associated with some degree of increased risk—at odds with the assumption underlying ongoing policy efforts that reducing benzodiazepine prescribing to long-term users will decrease harms. Future work is needed to examine mechanisms. It is possible that, having become physiologically dependent on benzodiazepine, patients experience adverse outcomes from withdrawal.^23^ Alternatively, patients may experience adverse consequences if they seek alternative sedating substances (eg, cannabis or alcohol) following benzodiazepine discontinuation.

These results were unexpected given findings from randomized clinical trials and reviews of benzodiazepine discontinuation. The EMPOWER (Eliminating Medications Through Patient Ownership of End Results) trial of patient education conducted 6-month follow-up interviews with participants, none of whom reported adverse events requiring hospitalization—although interviews could only be conducted with participants who completed the trial.^27^ In a 12-month primary care–based study, just 2 adverse events were reported among 542 participants.^28^ A meta-analysis of studies examining benzodiazepine discontinuation strategies captured whether included studies addressed adverse effects, but these were not actually reported in the meta-analysis.^34^ In a scoping review of deprescribing benzodiazepine, the only mention of potential adverse events was where they were related to other medications prescribed during the discontinuation process.^35^

There may be several reasons for the disagreement between our findings and prior studies. While emulated trials may address immortal time and survival biases, confounding may remain, whereby clinicians discontinue benzodiazepine treatment in patients otherwise at increased risk of death. However, there are other possible explanations. Our emulated trial followed patients for up to 1 year after discontinuation, which is longer than most randomized trials. Our inclusion criteria were more strict (ie, baseline year with 90% or more days covered; no coverage gaps longer than 30 consecutive days; monthly average daily dose within 30% of baseline grand mean) than those for randomized trials of benzodiazepine discontinuation, identifying a population potentially more likely to experience discontinuation-related distress and adverse effects. Patients willing to participate in a benzodiazepine discontinuation trial likely do not generalize to the broader population of those with stable long-term benzodiazepine use.

### Limitations

Our study has several limitations. Claims data cannot precisely determine when a patient stopped consuming their prescription benzodiazepine and patients may continue to receive medication from another source. Because benzodiazepine prescribing may predate plan enrollment, we cannot account for the full duration of patient benzodiazepine treatment, which may moderate the relationship between discontinuation and outcomes. While we employed multiple strategies to address bias in this trial emulation, unobserved confounding likely remains. Our analysis cannot account for prescribing indication or nature of the discontinuation process (eg, rate, adjunctive pharmacotherapy). Information was not available to identify which specific causes of death are elevated (eg, if discontinuers shifted to alternative substances with associated risks such as alcohol), which may have elucidated mechanisms. Finally, while this analysis includes a diverse population from across the US, it reflects those with commercial insurance or Medicare Advantage and thus may not generalize to other populations.

## Conclusions

In this emulated trial of benzodiazepine discontinuation among adults prescribed a stable long-term benzodiazepine regimen, there were small absolute increases in mortality and additional harms. Given the interest in reducing long-term prescribing, it will be important to conduct prospective studies or use observational approaches to replicate these findings and examine characteristics of patients and the discontinuation process that either attenuate or exacerbate this association. In addition, given questions raised about the safety of discontinuing benzodiazepine prescriptions among stable long-term users, clinicians should be judicious in initiating new prescriptions and carefully limit conversion to long-term use.

## Notes

Supplement 1. **eFigure.** Flowchart Presenting Cohort Identification **eTable 1.** Identification of Exclusion Criteria **eTable 2.** Diagnosis Codes Used to Identify Comorbidities **eTable 3.** American Hospital Formulary Service Codes for Medication Classes **eTable 4.** Outcome Definitions
eMethods.
**eTable 5.** Characteristics of Patients Prescribed Stable Long-term Benzodiazepine Therapy without Baseline Opioid Use at the End of the Grace Period, Overall and by Discontinuation Status: Before and After Weighting in the Intention-to-Treat Analysis **eTable 6.** Characteristics of Patients Prescribed Stable Long-term Benzodiazepine Therapy with Baseline Opioid Use at the End of the Grace Period, Overall and by Discontinuation Status: Before and After Weighting in the Intention-to-Treat Analysis **eTable 7.** Results of a Secondary Analysis Examining Benzodiazepine Discontinuation and Mortality Risk Among Patients Prescribed Stable Long-term Benzodiazepine Therapy, Stratified by Age, Intention-to-Treat Analysis **eTable 8.** Characteristics of Patients Prescribed Stable Long-term Benzodiazepine Therapy Stratified by Opioid Exposure at the End of the Grace Period for the Per-Protocol Analysis, Overall and by Benzodiazepine Discontinuation Status **eTable 9.** Characteristics of Patients Prescribed Stable Long-term Benzodiazepine Therapy without Opioid Exposure at the End of the Grace Period, Overall and by Discontinuation Status: Before and After Weighting in the Per-Protocol Analysis **eTable 10.** Characteristics of Patients Prescribed Stable Long-term Benzodiazepine Therapy with Opioid Exposure at the End of the Grace Period, Overall and by Discontinuation Status: Before and After Weighting in the Per-Protocol Analysis **eTable 11.** Adjusted Incidence, Risk Difference, and Risk Ratio of Mortality and Secondary Outcomes Among Patients Prescribed Stable Long-term Benzodiazepine Therapy, Stratified by Opioid Exposure, by Treatment Strategy **eTable 12.** Results of Sensitivity Analysis Examining Benzodiazepine Discontinuation and Mortality Risk Among Patients Prescribed Stable Long-term Benzodiazepine Therapy, Stratified by Opioid Use

Supplement 2.
Data Sharing Statement

## References

[1] Maust DT, Lin LA, Blow FC. Benzodiazepine use and misuse among adults in the United States. Psychiatr Serv. 2019;70(2):97-106. doi:10.1176/appi.ps.201800321 30554562 PMC6358464

[2] Bachhuber MA, Hennessy S, Cunningham CO, Starrels JL. Increasing benzodiazepine prescriptions and overdose mortality in the United States, 1996-2013. Am J Public Health. 2016;106(4):686-688. doi:10.2105/AJPH.2016.303061 26890165 PMC4816010

[3] Jones CM, Mack KA, Paulozzi LJ. Pharmaceutical overdose deaths, United States, 2010. JAMA. 2013;309(7):657-659. doi:10.1001/jama.2013.272 23423407

[4] National Institute on Drug Abuse, US Department of Health and Human Services. National Drug Overdose Deaths Involving Benzodiazepines, by Opioid Involvement, Number Among All Ages, 1999-2020. Accessed August 4, 2022. https://nida.nih.gov/research-topics/trends-statistics/overdose-death-rates#:~:text=Drug%20overdose%20deaths%20involving%20benzodiazepines%20has%20steadily%20increased%20from%201%2C135,and%20rose%20again%20to%2012%2C290

[5] US Food and Drug Administration. FDA Drug Safety Communication: FDA warns about serious risks and death when combining opioid pain or cough medicines with benzodiazepines; requires its strongest warning. August 31, 2016. Accessed May 10, 2019. https://www.fda.gov/media/99761/download

[6] U.S. Food & Drug Administration. FDA Drug Safety Communication: FDA requiring Boxed Warning updated to improve safe use of benzodiazepine drug class. September 23, 2020. Accessed October 15, 2020. https://www.fda.gov/media/142368/download

[7] US Food and Drug Administration. Cooperative Agreement to Support an Evidence-Based Clinical Practice Guideline for the Safe Tapering of Benzodiazepines (U01) Clinical Trials Not Allowed. Posted May 31, 2022. Accessed August 11, 2023. https://grants.nih.gov/grants/guide/rfa-files/RFA-FD-22-027.html

[8] Soyka M. Treatment of Benzodiazepine Dependence. N Engl J Med. 2017;376(12):1147-1157. doi:10.1056/NEJMra1611832 28328330

[9] Lembke A, Papac J, Humphreys K. Our other prescription drug problem. N Engl J Med. 2018;378(8):693-695. doi:10.1056/NEJMp1715050 29466163

[10] Baldwin DS, Anderson IM, Nutt DJ, . Evidence-based pharmacological treatment of anxiety disorders, post-traumatic stress disorder and obsessive-compulsive disorder: a revision of the 2005 guidelines from the British Association for Psychopharmacology. J Psychopharmacol. 2014;28(5):403-439. doi:10.1177/0269881114525674 24713617

[11] Wagner AK, Zhang F, Soumerai SB, . Benzodiazepine use and hip fractures in the elderly: who is at greatest risk? Arch Intern Med. 2004;164(14):1567-1572. doi:10.1001/archinte.164.14.1567 15277291

[12] Woolcott JC, Richardson KJ, Wiens MO, . Meta-analysis of the impact of 9 medication classes on falls in elderly persons. Arch Intern Med. 2009;169(21):1952-1960. doi:10.1001/archinternmed.2009.357 19933955

[13] Rapoport MJ, Lanctôt KL, Streiner DL, . Benzodiazepine use and driving: a meta-analysis. J Clin Psychiatry. 2009;70(5):663-673. doi:10.4088/JCP.08m04325 19389334

[14] Park TW, Saitz R, Ganoczy D, Ilgen MA, Bohnert AS. Benzodiazepine prescribing patterns and deaths from drug overdose among US veterans receiving opioid analgesics: case-cohort study. BMJ. 2015;350:h2698. doi:10.1136/bmj.h2698 26063215 PMC4462713

[15] Larochelle MR, Lodi S, Yan S, Clothier BA, Goldsmith ES, Bohnert ASB. Comparative effectiveness of opioid tapering or abrupt discontinuation vs no dosage change for opioid overdose or suicide for patients receiving stable long-term opioid therapy. JAMA Netw Open. 2022;5(8):e2226523. doi:10.1001/jamanetworkopen.2022.26523 35960518 PMC9375167

[16] James JR, Scott JM, Klein JW, . Mortality after discontinuation of primary care–based chronic opioid therapy for pain: a retrospective cohort study. J Gen Intern Med. 2019;34(12):2749-2755. doi:10.1007/s11606-019-05301-2 31468341 PMC6854174

[17] Mark TL, Parish W. Opioid medication discontinuation and risk of adverse opioid-related health care events. J Subst Abuse Treat. 2019;103:58-63. doi:10.1016/j.jsat.2019.05.001 31079950

[18] Oliva EM, Bowe T, Manhapra A, . Associations between stopping prescriptions for opioids, length of opioid treatment, and overdose or suicide deaths in US veterans: observational evaluation. BMJ. 2020;368:m283. doi:10.1136/bmj.m283 32131996 PMC7249243

[19] Hallvik SE, El Ibrahimi S, Johnston K, . Patient outcomes after opioid dose reduction among patients with chronic opioid therapy. Pain. 2022;163(1):83-90. doi:10.1097/j.pain.0000000000002298 33863865 PMC8494834

[20] Cook JM, Biyanova T, Masci C, Coyne JC. Older patient perspectives on long-term anxiolytic benzodiazepine use and discontinuation: a qualitative study. J Gen Intern Med. 2007;22(8):1094-1100. doi:10.1007/s11606-007-0205-5 17492325 PMC2305752

[21] Fixsen AM. “I’m not waving, I’m drowning”: an autoethnographical exploration of biographical disruption and reconstruction during recovery from prescribed benzodiazepine use. Qual Health Res. 2016;26(4):466-481. doi:10.1177/1049732315576496 25800715

[22] Ashton H. The diagnosis and management of benzodiazepine dependence. Curr Opin Psychiatry. 2005;18(3):249-255. doi:10.1097/01.yco.0000165594.60434.84 16639148

[23] Pétursson H. The benzodiazepine withdrawal syndrome. Addiction. 1994;89(11):1455-1459. doi:10.1111/j.1360-0443.1994.tb03743.x 7841856

[24] Hernán MA, Robins JM. Using big data to emulate a target trial when a randomized trial is not available. Am J Epidemiol. 2016;183(8):758-764. doi:10.1093/aje/kwv254 26994063 PMC4832051

[25] Galanter M, Kleber HD. The American Psychiatric Publishing textbook of substance abuse treatment. 4th ed. American Psychiatric Pub; 2008.

[26] Belviso NJ. The Comparative Effectiveness, Safety, and Cost of Oral P2Y12 Antiplatelet Agents Following Acute Coronary Syndromes. University of Rhode Island; 2020.

[27] Tannenbaum C, Martin P, Tamblyn R, Benedetti A, Ahmed S. Reduction of inappropriate benzodiazepine prescriptions among older adults through direct patient education: the EMPOWER cluster randomized trial. JAMA Intern Med. 2014;174(6):890-898. doi:10.1001/jamainternmed.2014.949 24733354

[28] Vicens C, Bejarano F, Sempere E, . Comparative efficacy of two interventions to discontinue long-term benzodiazepine use: cluster randomised controlled trial in primary care. Br J Psychiatry. 2014;204(6):471-479. doi:10.1192/bjp.bp.113.134650 24526745

[29] Voshaar RC, Gorgels WJ, Mol AJ, . Tapering off long-term benzodiazepine use with or without group cognitive-behavioural therapy: three-condition, randomised controlled trial. Br J Psychiatry. 2003;182:498-504. doi:10.1192/bjp.182.6.498 12777340

[30] Gorgels WJ, Oude Voshaar RC, Mol AJ, . Predictors of discontinuation of benzodiazepine prescription after sending a letter to long-term benzodiazepine users in family practice. Fam Pract. 2006;23(1):65-72. doi:10.1093/fampra/cmi065 16107495

[31] Morin CM, Bastien C, Guay B, Radouco-Thomas M, Leblanc J, Vallières A. Randomized clinical trial of supervised tapering and cognitive behavior therapy to facilitate benzodiazepine discontinuation in older adults with chronic insomnia. Am J Psychiatry. 2004;161(2):332-342. doi:10.1176/appi.ajp.161.2.332 14754783

[32] Danaei G, García Rodríguez LA, Cantero OF, Logan RW, Hernán MA. Electronic medical records can be used to emulate target trials of sustained treatment strategies. J Clin Epidemiol. 2018;96:12-22. doi:10.1016/j.jclinepi.2017.11.021 29203418 PMC5847447

[33] Sun EC, Dixit A, Humphreys K, Darnall BD, Baker LC, Mackey S. Association between concurrent use of prescription opioids and benzodiazepines and overdose: retrospective analysis. BMJ. 2017;356:j760. doi:10.1136/bmj.j760 28292769 PMC5421443

[34] Voshaar RCO, Couvée JE, van Balkom AJ, Mulder PG, Zitman FG. Strategies for discontinuing long-term benzodiazepine use: meta-analysis. Br J Psychiatry. 2006;189(3):213-220. doi:10.1192/bjp.189.3.213 16946355

[35] Pollmann AS, Murphy AL, Bergman JC, Gardner DM. Deprescribing benzodiazepines and Z-drugs in community-dwelling adults: a scoping review. BMC Pharmacol Toxicol. 2015;16:19. doi:10.1186/s40360-015-0019-8 26141716 PMC4491204

