# Supplementary material for: Benzodiazepine Discontinuation and Mortality Among Patients Receiving Long-Term Benzodiazepine Therapy
Source: JAMA Netw Open. 2023 Dec 20;6(12):e2348557. doi: 10.1001/jamanetworkopen.2023.48557 (PMC10733804; doi:10.1001/jamanetworkopen.2023.48557)
Supplement: Supplement 1. — eFigure. Flowchart Presenting Cohort Identification eTable 1. Identification of Exclusion Criteria eTable 2. Diagnosis Codes Used to Identify Comorbidities eTable 3. American Hospital Formulary Service Codes for Medication Classes eTable 4. Outcome Definitions eMethods. eTable 5. Characteristics of Patients Prescribed Stable Long-term Benzodiazepine Therapy without Baseline Opioid Use at the End of the Grace Period, Overall and by Discontinuation Status: Before and After Weighting in the Intention-to-Treat Analysis eTable 6. Characteristics of Patients Prescribed Stable Long-term Benzodiazepine Therapy with Baseline Opioid Use at the End of the Grace Period, Overall and by Discontinuation Status: Before and After Weighting in the Intention-to-Treat Analysis eTable 7. Results of a Secondary Analysis Examining Benzodiazepine Discontinuation and Mortality Risk Among Patients Prescribed Stable Long-term Benzodiazepine Therapy, Stratified by Age, Intention-to-Treat Analysis eTable 8. Characteristics of Patients Prescribed Stable Long-term Benzodiazepine Therapy Stratified by Opioid Exposure at the End of the Grace Period for the Per-Protocol Analysis, Overall and by Benzodiazepine Discontinuation Status eTable 9. Characteristics of Patients Prescribed Stable Long-term Benzodiazepine Therapy without Opioid Exposure at the End of the Grace Period, Overall and by Discontinuation Status: Before and After Weighting in the Per-Protocol Analysis eTable 10. Characteristics of Patients Prescribed Stable Long-term Benzodiazepine Therapy with Opioid Exposure at the End of the Grace Period, Overall and by Discontinuation Status: Before and After Weighting in the Per-Protocol Analysis eTable 11. Adjusted Incidence, Risk Difference, and Risk Ratio of Mortality and Secondary Outcomes Among Patients Prescribed Stable Long-term Benzodiazepine Therapy, Stratified by Opioid Exposure, by Treatment Strategy eTable 12. Results of Sensitivity Analysis Examining Benzodiazepine Discontinuation a [file jamanetwopen-e2348557-s001.pdf]

Supplemental Online Content

Maust DT, Petzold K, Strominger J, Kim HM, Bohnert AB. Benzodiazepine discontinuation and mortality among patients on long-term benzodiazepine therapy. *JAMA Netw Open.* 2023;6(12):e2348557. doi:10.1001/jamanetworkopen.2023.48557

eFigure. Flowchart Presenting Cohort Identification

eTable 1. Identification of Exclusion Criteria

eTable 2. Diagnosis Codes Used to Identify Comorbidities

eTable 3. American Hospital Formulary Service Codes for Medication Classes

eTable 4. Outcome Definitions

eMethods.

eTable 5. Characteristics of Patients Prescribed Stable Long-term Benzodiazepine Therapy without Baseline Opioid Use at the End of the Grace Period, Overall and by Discontinuation Status: Before and After Weighting in the Intention-to-Treat Analysis

eTable 6. Characteristics of Patients Prescribed Stable Long-term Benzodiazepine Therapy with Baseline Opioid Use at the End of the Grace Period, Overall and by Discontinuation Status: Before and After Weighting in the Intention-to-Treat Analysis

eTable 7. Results of a Secondary Analysis Examining Benzodiazepine Discontinuation and Mortality Risk Among Patients Prescribed Stable Long-term Benzodiazepine Therapy, Stratified by Age, Intention-to-Treat Analysis

eTable 8. Characteristics of Patients Prescribed Stable Long-term Benzodiazepine Therapy Stratified by Opioid Exposure at the End of the Grace Period for the Per-Protocol Analysis, Overall and by Benzodiazepine Discontinuation Status

eTable 9. Characteristics of Patients Prescribed Stable Long-term Benzodiazepine Therapy without Opioid Exposure at the End of the Grace Period, Overall and by Discontinuation Status: Before and After Weighting in the Per-Protocol Analysis

eTable 10. Characteristics of Patients Prescribed Stable Long-term Benzodiazepine Therapy with Opioid Exposure at the End of the Grace Period, Overall and by Discontinuation Status: Before and After Weighting in the Per-Protocol Analysis

eTable 11. Adjusted Incidence, Risk Difference, and Risk Ratio of Mortality and Secondary Outcomes Among Patients Prescribed Stable Long-term Benzodiazepine Therapy, Stratified by Opioid Exposure, By Treatment Strategy

© 2023 Maust DT et al. *JAMA Network Open.*

eTable 12. Results of Sensitivity Analysis Examining Benzodiazepine Discontinuation and Mortality Risk Among Patients Prescribed Stable Long-term Benzodiazepine Therapy, Stratified by Opioid Use

This supplemental material has been provided by the authors to give readers additional information about their work.

© 2023 Maust DT et al. *JAMA Network Open.*

Figure S1. Flowchart Presenting Cohort Identification

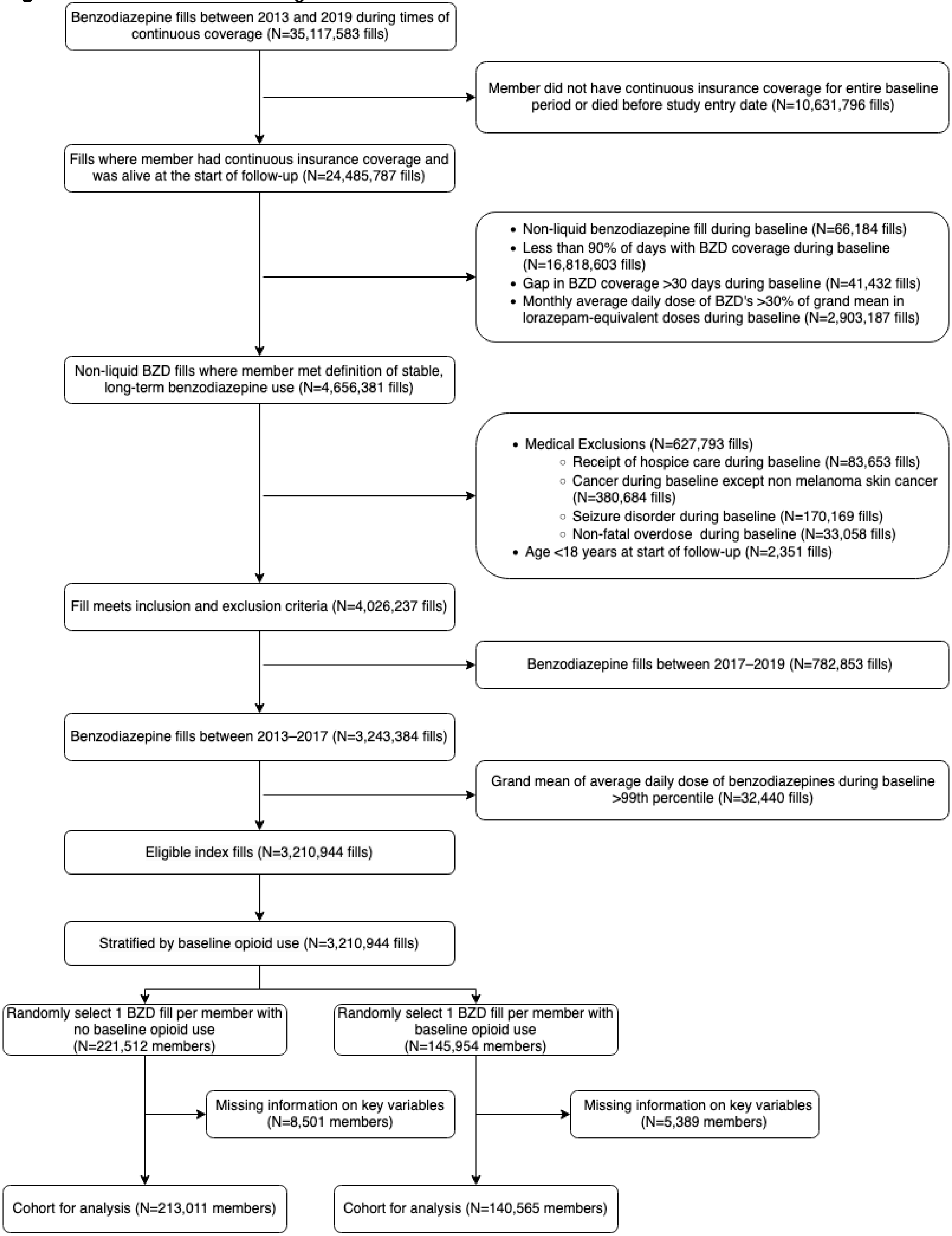

© 2023 Maust DT et al. *JAMA Network Open.*

Table e1. Identification of Exclusion Criteria

Exclusion	Source	Codes
Receipt of hospice care	Revenue	115, 0125, 0135, 0145, 0155, 0235, 0650, 0651, 0652, 0653, 0654, 0655, 0656, 0657, 0658, 0659
	HCPCS	99377, 99378, G0182, G0337, G9474, G9475, G9476, G9477, G9478, G9479, G9524, Q5001, Q5002, Q5003, Q5004, Q5005, Q5006, Q5007, Q5008, Q5009, Q5010, S0255, S9126, T2042, T2043, T2044, T2045, T2046
Cancer except nonmelanoma skin cancer	ICD-9	140.x-208.x(except 173.x), 209.0-209.3, V10.x (except V10.83)
	ICD-10	C00.x-C80.x (except C44.x, include C7A.x, exclude C7B.x)
Seizure disorder	ICD-9	345. (i.e., any diagnosis code that start with 345)
	ICD-10	G40. (i.e., any diagnosis code that starts with G40)
Non-fatal overdose (identified at emergency room/hospitalization visits)	ICD-9	960-979, E850-E858, E950.0-E950.5, E980.0-E980.5, E962.0
	ICD-10	T36-T50, where 5th/6th character = 1-4 and 7th char = A.

© 2023 Maust DT et al. *JAMA Network Open.*

Table e2. Diagnosis Codes Used to Identify Comorbidities

Comorbidity	Source	Codes
Anxiety	ICD-9	300, 300.0, 300.00-300.02, 300.09, 300.10, 300.2, 300.20-300.23, 300.29 309.81
	ICD-10	F40.x, F41.x, F43.10-F43.12
Major depression	ICD-9	296.2x, 296.3x
	ICD-10	F32, F32.0, F32.1, F32.2, F32.3, F32.4, F32.5, F32.9, F33, F33.0, F33.1, F33.2, F33.3, F33.4, F33.40, F33.41, F33.42, F33.8, F33.9
Chronic pain	ICD-9	256.60, 307.80, 307.81, 307.89, 346.0-346.9, 355.0, 355.9, 356.0, 357.2, 357.9, 710.0-739.9, 784.0
	ICD-10	We used the following ICD-9-CM non-cancer chronic pain scheme, after Ilgen et al.[1]<br>• back pain (720.0-724.9)<br>• arthritis (710.0-739.9, excluding back pain codes)<br>• migraines (346.0-346.9)<br>• headache or tension headache (784.0 and 307.81)<br>• psychogenic pain (307.80 and 307.89)<br>• neuropathy (256.60, 355.0, 355.9, 356.0, 357.2, and 357.9)<br>• fibromyalgia (729.1)<br><br>We used the ICD-9 to -10 crosswalk from CMS2 to identify the equivalent ICD-10 codes. The full list of equivalent ICD-10 codes is available from the authors.
Bipolar	ICD-9	296, 296.0, 296.00-296.06, 296.1, 296.10-296.16, 296.4, 296.40-296.46, 296.5, 296.50-296.56, 296.6, 296.60-296.66, 296.7, 296.70, 296.75, 296.8, 296.80-296.83, 296.89, 301.13
	ICD-10	F30.10-F30.13, F30.2-F30.4, F30.8, F30.9, F31*, F34.0
Psychotic disorders	ICD-9	295.x, 297.x, 298.1-298.9, 301.22
	ICD-10	F20.x, F21.x, F22.x, F23.x, F24.x, F25.x, F28.x, F29.x
Insomnia	ICD-9	307.4x, 327.00, 327.01, 327.02, 327.09, 780.51, 780.52
	ICD-10	G47.00. G47.01. G47.09
Substance use disorder: alcohol	ICD-9	303.0x, 303.9x, 305.0x
	ICD-10	F10.1X, F10.2X, F10.9x
Substance use disorder: nonalcohol		
Opioid	ICD-9	304.0x, 304.7x, 305.5x, excluding 304.03,304.73, 305.53
	ICD-10	F11.1X, F11.2X
Cocaine/Stimulants	ICD-9	304.2x, 304.4x, 305.6x, 305.7x
	ICD-10	F14.1X, F14.2X, F14.9X, F15.1X, F15.2X, F15.9X
Sedatives	ICD-9	304.1x, 305.4x
	ICD-10	F13.1X, F13.2X, F13.9X
Cannabis	ICD-9	304.3x, 305.2x
	ICD-10	F12.1X, F12.2X, F12.9X
Other	ICD-9	304.5x, 304.6x, 304.8x, 304.9x, 305.3x, 305.8x, 305.9x
	ICD-10	F16.1X, F16.2X, F16.9X, F18.1X, F18.2X, F18.9X, F19.1X, F19.2X, F19.9X

[1] Ilgen MA, Kleinberg F, Ignacio RV, Bohnert ASB, Valenstein M, McCarthy JF, *et al.* Noncancer pain conditions and risk of suicide. *JAMA Psychiatry* 2013;**70**:692–7.

© 2023 Maust DT et al. *JAMA Network Open.*

Table e3. American Hospital Formulary Service (AHFS) Codes for Medication Classes

Medication class	AHFS codes
Benzodiazepine	28:12.08, 28:24.08
Antidepressants	28:16.04.xx
Antiepileptics	28:12.xx (except 28:12.08)
Antipsychotics	28:16.08.xx
Z-drugs[a]	28:24.92

[a] Includes other non-benzodiazepine anxiolytic/sedative-hypnotics (e.g., buspirone, chloral hydrate)

© 2023 Maust DT et al. *JAMA Network Open.*

Table e4. Outcome Definitions

Outcome	Source	Codes
All-cause mortality		Date of death provided as month/year, randomized day of death within month/year using uniform distribution
Non-fatal overdose (identified at emergency room /hospitalization visits)	ICD-9	960-979, E850-E858, E950.0-E950.5, E980.0-E980.5, E962.0
	ICD-10	T36-T50, where 5th/6th character = 1, 2, 4, or 5 and 7th char = A.
Suicide attempts and self-inflicted injury	ICD-9	E950-E958, E959
	ICD-10	X71-X83, T36-T50 5th/6th char=2, T51-T65 with 5th/6th char - 2, T71 with 6th char = 2, and T14.91
Emergency department use	HCPCS	99281-99285 or 99291 where POS = 23
	Revenue	0450-0452, 0456, 0459, 0981 (Inpatient or outpatient)
Suicidal ideation	ICD-9	V62.84
	ICD-10	R45.851

© 2023 Maust DT et al. *JAMA Network Open.*

Supplementary Methods. Additional Information Regarding Construction of Weights

We provide additional information regarding construction of inverse probability of censoring weights for the main analysis. After cloning a given stratified dataset (e.g., stable, long-term benzodiazepine [BZD] users without baseline opioid use), we created 3 sets of time-varying weights:

1) a treatment assignment weight to account for potential informative censoring of clones,

2) a loss-to-follow-up weight to account for insurance disenrollment, and

3) a weight to account for nonadherence to the assigned treatment arm (i.e., *discontinued BZD* and *did not discontinue BZD*). The first two weights are used in the intention-to-treat analysis whereas all three weights are used in the per-protocol analysis.

The first weight to account for potential informative censoring of clones during the grace period was derived by first constructing a clone-month dataset where the outcome was set to 1 if the given clone was censored due to treatment assignment in the given month and 0 if the clone was not censored due to treatment assignment in the given month. For example, if person A discontinued their BZD in month 2, person A's clone in the *did not discontinue* treatment arm was censored due to treatment assignment in month 2. Because treatment is assigned during the grace period, censoring due to treatment assignment can only occur during the grace period. Clones in the *did not discontinue* treatment arm can be censored due to treatment assignment as early as month 2 because our exposure, BZD discontinuation, is defined as 31 consecutive days of no BZD coverage (and we define one month as a 30-day period). Clones in the *discontinue* BZD treatment arm were censored due to treatment assignment only in month 6 (i.e., at the end of the grace period) for those who did not discontinue BZD during the grace period. Using the cloned dataset and stratifying by treatment arm, we then fit a pooled logistic regression model where the outcome was not being censored in the given month due to treatment assignment; covariates included baseline confounders and time (included as linear, quadratic, and cubic terms). We then computed the predicted probability of not being censored for each clone-month and took the inverse to create a weight for each clone-month. If the clone was censored due to treatment assignment in the given month, the weight was set to 0 for the month and all subsequent months. For clones that had not been censored during the grace period due to treatment assignment, we set the weight to 1 for all months post-grace period. We then computed a final weight for treatment assignment for each clone-month by taking the cumulative product of the monthly weights.

The same steps were repeated to create two additional weights that account for loss-to-follow-up and nonadherence after assignment to a single treatment arm. A given clone could have loss-to-follow-up (i.e., insurance disenrollment) any month during follow-up. For nonadherence, a given clone could only be nonadherent after the given person is assigned to a single treatment arm (i.e., a person must be assigned to one treatment arm before they can be nonadherent). Therefore, for persons assigned to the *did not discontinue* BZD arm, nonadherence could only occur after the grace period. For the *discontinued* BZD arm, nonadherence could occur as early as month 2 of follow-up because a given person could be assigned to the *discontinued* BZD arm as early as month 2 but then also fill a BZD later during that same month.

After computing inverse probability of censoring weights to account for potential informative censoring of clones, loss-to-follow-up (i.e., insurance disenrollment), and nonadherence to the assigned treatment, we constructed final weights for the intention-to-treat and per-protocol analyses. The final weight for the intention-to-treat analysis was computed using the clone-month dataset and by multiplying the weights that account for potential informative censoring of clones during the grace period and insurance disenrollment. The final weight for the per-protocol analysis is the same except that we additionally multiply by the weight that accounts for nonadherence after treatment assignment. Prior to fitting the analytic model, we truncated weights at the 99th percentile.

All steps were followed, separately, for those without and with baseline opioid use. The same approach was used for secondary outcomes except that when computing the weight to account for loss-to-follow-up, we also accounted for death (i.e., the primary outcome).

© 2023 Maust DT et al. *JAMA Network Open.*

Table e5. Characteristics of Patients Prescribed Stable Long-term Benzodiazepine Therapy **without** Opioid Exposure[a] at the End of the Grace Period, Overall and by Discontinuation Status[b]: Before and after Weighting in the **Intention-to-Treat** Analysis

	Unweighted			Weighted		
**Characteristic, N (%) or mean (SD)**	**Overall**	**Discontinued**	**Not Discontinued**	**Overall**	**Discontinued**	**Not Discontinued**
**N**	178934 (100)	38010 (21.2)	140924 (78.8)	394952 (100)	187292 (47.4)	207661 (52.5)
Age						
18-44	20808 (11.6)	4740 (12.5)	16068 (11.4)	51909 (13.1)	24238 (12.9)	27671 (13.3)
45-64	67303 (37.6)	13437 (35.4)	53866 (38.2)	154108 (39.0)	71824 (38.3)	82285 (39.6)
65+	90823 (50.8)	19833 (52.2)	70990 (50.4)	188935 (47.8)	91230 (48.7)	97705 (47.1)
Female	115539 (64.6)	24360 (64.1)	91179 (64.7)	253379 (64.2)	120255 (64.2)	133124 (64.1)
Race/ethnicity						
Asian	2506 (1.4)	601 (1.6)	1905 (1.4)	5530 (1.4)	2655 (1.4)	2875 (1.4)
Black	16117 (9.0)	3361 (8.8)	12756 (9.1)	35311 (8.9)	16845 (9.0)	18465 (8.9)
Hispanic	19589 (10.9)	4317 (11.4)	15272 (10.8)	42833 (10.8)	20610 (11.0)	22223 (10.7)
White	140722 (78.6)	29731 (78.2)	110991 (78.8)	311279 (78.8)	147182 (78.6)	164097 (79.0)
Region						
Midwest	36774 (20.6)	7277 (19.1)	29497 (20.9)	82316 (20.8)	38398 (20.5)	43917 (21.1)
Northeast	17399 (9.7)	3277 (8.6)	14122 (10.0)	38373 (9.7)	17818 (9.5)	20555 (9.9)
South	91606 (51.2)	19240 (50.6)	72366 (51.4)	202292 (51.2)	96140 (51.3)	106152 (51.1)
West	33155 (18.5)	8216 (21.6)	24939 (17.7)	71971 (18.2)	34936 (18.7)	37036 (17.8)
Anxiety	91973 (51.4)	18805 (49.5)	73168 (51.9)	204246 (51.7)	96191 (51.4)	108055 (52.0)
Depression	50304 (28.1)	10623 (27.9)	39681 (28.2)	110178 (27.9)	52202 (27.9)	57976 (27.9)
Chronic pain	128479 (71.8)	27902 (73.4)	100577 (71.4)	282214 (71.5)	134476 (71.8)	147738 (71.1)
Insomnia	29841 (16.7)	6985 (18.4)	22856 (16.2)	66826 (16.9)	31993 (17.1)	34833 (16.8)
Bipolar	14834 (8.3)	2973 (7.8)	11861 (8.4)	32788 (8.3)	15340 (8.2)	17449 (8.4)
Other psychotic disorders	6968 (3.9)	1615 (4.2)	5353 (3.8)	15167 (3.8)	7220 (3.9)	7947 (3.8)
Substance use disorders						
Alcohol	4554 (2.5)	1171 (3.1)	3383 (2.4)	10542 (2.7)	5127 (2.7)	5414 (2.6)
Opioid	4810 (2.7)	1432 (3.8)	3378 (2.4)	10913 (2.8)	5300 (2.8)	5613 (2.7)
Stimulant	806 (0.5)	265 (0.7)	541 (0.4)	1865 (0.5)	912 (0.5)	953 (0.5)
Sedative	4602 (2.6)	1060 (2.8)	3542 (2.5)	9808 (2.5)	4746 (2.5)	5062 (2.4)
Cannabis	1143 (0.6)	325 (0.9)	818 (0.6)	2727 (0.7)	1333 (0.7)	1395 (0.7)
Other substances	2401 (1.3)	712 (1.9)	1689 (1.2)	5604 (1.4)	2704 (1.4)	2900 (1.4)
Modified Elixhauser score[c]	2.67 (2.41)	2.79 (2.55)	2.63 (2.37)	2.60 (3.57)	2.61 (5.44)	2.58 (2.87)
Average BZD lor-eq mg/day	2.75 (2.48)	2.47 (2.36)	2.83 (2.50)	2.74 (3.72)	2.67 (5.63)	2.80 (2.99)
30-day medication prescriptions						
Antidepressants	8.02 (8.81)	7.79 (8.57)	8.09 (8.87)	7.97 (12.97)	7.94 (19.22)	8.00 (10.68)
Antiepileptics	2.72 (5.65)	2.69 (5.54)	2.73 (5.68)	2.69 (8.31)	2.68 (12.32)	2.69 (6.84)
Antipsychotics	1.73 (5.00)	1.56 (4.68)	1.77 (5.08)	1.69 (7.31)	1.65 (10.74)	1.72 (6.06)
Z-drugs	0.86 (2.94)	0.81 (2.81)	0.87 (2.97)	0.89 (4.43)	0.87 (6.49)	0.90 (3.68)
Year (at start of follow-up)						
2014	27475 (15.4)	6743 (17.7)	20732 (14.7)	65962 (16.7)	31395 (16.8)	34567 (16.6)
2015	27191 (15.2)	6568 (17.3)	20623 (14.6)	60519 (15.3)	28948 (15.5)	31571 (15.2)
2016	29595 (16.5)	6772 (17.8)	22823 (16.2)	66425 (16.8)	31609 (16.9)	34816 (16.8)
2017	37941 (21.2)	7750 (20.4)	30191 (21.4)	82810 (21.0)	39151 (20.9)	43659 (21.0)
2018	56732 (31.7)	10177 (26.8)	46555 (33.0)	119236 (30.2)	56188 (30.0)	63048 (30.4)

SD: standard deviation; BZD: benzodiazepine; lor-eq: lorazepam-equivalent
[a] Opioid exposure is defined as the presence of at least one prescription opioid fill during the last 30 days of the baseline period.
[b] Discontinuation is defined as 31 consecutive days without prescription benzodiazepine coverage. Characteristics determined at end of the grace period, by which time each patient has a maximum of one clone remaining.
[c] The Elixhauser score is modified to exclude depression, substance abuse, alcohol abuse, and psychosis as these are included as separate covariates.

© 2023 Maust DT et al. *JAMA Network Open.*

Table e6. Characteristics of Patients Prescribed Stable Long-term Benzodiazepine Therapy **with** Opioid Exposure[a] at the End of the Grace Period, Overall and by Discontinuation Status[b]: Before and after Weighting in the **Intention-to-Treat** Analysis

	**Unweighted**			**Weighted**		
**Characteristic, N (%) or mean (SD)**	**Overall**	**Discontinued**	**Not Discontinued**	**Overall**	**Discontinued**	**Not Discontinued**
**N**	118919 (100)	22287 (18.7)	96632 (81.3)	257550 (100)	120553 (46.8)	136996 (53.2)
Age						
18-44	11643 (9.8)	2256 (10.1)	9387 (9.7)	27840 (10.8)	12684 (10.5)	15155 (11.1)
45-64	56562 (47.6)	10096 (45.3)	46466 (48.1)	124516 (48.3)	57436 (47.6)	67080 (49.0)
65+	50714 (42.6)	9935 (44.6)	40779 (42.2)	105194 (40.8)	50433 (41.8)	54761 (40.0)
Female	78001 (65.6)	14433 (64.8)	63568 (65.8)	167824 (65.2)	78312 (65.0)	89512 (65.3)
Race/ethnicity						
Asian	1100 (0.9)	223 (1.0)	877 (0.9)	2351 (0.9)	1089 (0.9)	1262 (0.9)
Black	13638 (11.5)	2671 (12.0)	10967 (11.3)	29517 (11.5)	13997 (11.6)	15521 (11.3)
Hispanic	10389 (8.7)	2067 (9.3)	8322 (8.6)	22586 (8.8)	10838 (9.0)	11748 (8.6)
White	93792 (78.9)	17326 (77.7)	76466 (79.1)	203096 (78.9)	94630 (78.5)	108466 (79.2)
Region						
Midwest	22708 (19.1)	3834 (17.2)	18874 (19.5)	49669 (19.3)	22578 (18.7)	27091 (19.8)
Northeast	8550 (7.2)	1346 (6.0)	7204 (7.5)	18331 (7.1)	8236 (6.8)	10095 (7.4)
South	65751 (55.3)	12410 (55.7)	53341 (55.2)	142790 (55.4)	67235 (55.8)	75556 (55.2)
West	21910 (18.4)	4697 (21.1)	17213 (17.8)	46760 (18.2)	22505 (18.7)	24254 (17.7)
Anxiety	67578 (56.8)	12230 (54.9)	55348 (57.3)	146565 (56.9)	68057 (56.5)	78508 (57.3)
Depression	37339 (31.4)	7052 (31.6)	30287 (31.3)	80015 (31.1)	37490 (31.1)	42524 (31.0)
Chronic pain	107289 (90.2)	20533 (92.1)	86756 (89.8)	233204 (90.5)	109592 (90.9)	123612 (90.2)
Insomnia	21887 (18.4)	4643 (20.8)	17244 (17.8)	48306 (18.8)	22878 (19.0)	25428 (18.6)
Bipolar	10556 (8.9)	1849 (8.3)	8707 (9.0)	22582 (8.8)	10407 (8.6)	12176 (8.9)
Other psychotic disorders	3968 (3.3)	775 (3.5)	3193 (3.3)	8543 (3.3)	4002 (3.3)	4541 (3.3)
Substance use disorders						
Alcohol	3416 (2.9)	711 (3.2)	2705 (2.8)	7572 (2.9)	3569 (3.0)	4003 (2.9)
Opioid	10163 (8.5)	2316 (10.4)	7847 (8.1)	22527 (8.7)	10760 (8.9)	11767 (8.6)
Stimulant	770 (0.6)	194 (0.9)	576 (0.6)	1781 (0.7)	855 (0.7)	926 (0.7)
Sedative	2941 (2.5)	617 (2.8)	2324 (2.4)	6388 (2.5)	3103 (2.6)	3286 (2.4)
Cannabis	1008 (0.8)	228 (1.0)	780 (0.8)	2263 (0.9)	1070 (0.9)	1194 (0.9)
Other substances	3531 (3.0)	832 (3.7)	2699 (2.8)	8068 (3.1)	3854 (3.2)	4214 (3.1)
Modified Elixhauser score[c]	3.17 (2.57)	3.36 (2.71)	3.13 (2.53)	3.13 (3.78)	3.16 (6.04)	3.11 (3.02)
Average BZD lor-eq mg/day	3.47 (2.87)	3.03 (2.69)	3.57 (2.90)	3.43 (4.23)	3.33 (6.73)	3.52 (3.40)
30-day medication prescriptions						
Antidepressants	8.76 (9.21)	8.55 (9.10)	8.80 (9.24)	8.65 (13.46)	8.60 (21.19)	8.69 (10.93)
Antiepileptics	3.90 (6.35)	4.03 (6.35)	3.86 (6.36)	3.88 (9.28)	3.91 (14.57)	3.86 (7.56)
Antipsychotics	1.61 (4.62)	1.43 (4.31)	1.65 (4.69)	1.57 (6.69)	1.54 (10.44)	1.60 (5.47)
Z-drugs	1.19 (3.42)	1.16 (3.31)	1.20 (3.44)	1.22 (5.07)	1.21 (7.89)	1.24 (4.16)
Year (at start of follow-up)						
2014	20211 (17.0)	4001 (18.0)	16210 (16.8)	46781 (18.2)	21645 (18.0)	25136 (18.3)
2015	18861 (15.9)	3467 (15.6)	15394 (15.9)	40744 (15.8)	19020 (15.8)	21724 (15.9)
2016	20942 (17.6)	3852 (17.3)	17090 (17.7)	45408 (17.6)	21122 (17.5)	24286 (17.7)
2017	27907 (23.5)	5004 (22.5)	22903 (23.7)	59630 (23.2)	27859 (23.1)	31771 (23.2)
2018	30998 (26.1)	5963 (26.8)	25035 (25.9)	64986 (25.2)	30907 (25.6)	34079 (24.9)

SD: standard deviation; BZD: benzodiazepine; lor-eq: lorazepam-equivalent

[a] Opioid exposure is defined as the presence of at least one prescription opioid fill during the last 30 days of the baseline period.

[b] Discontinuation is defined as 31 consecutive days without prescription benzodiazepine coverage. Characteristics determined at end of the grace period, by which time each patient has a maximum of one clone remaining.

[c] The Elixhauser score is modified to exclude depression, substance abuse, alcohol abuse, and psychosis as these are included as separate covariates.

© 2023 Maust DT et al. *JAMA Network Open.*

Table e7. Results of a Secondary Analysis Examining Benzodiazepine Discontinuation and Mortality Risk Among Patients Prescribed Stable Long-term Benzodiazepine Therapy, Stratified by Age, Intention-to-Treat Analysis

	%			Risk ratio
	Adjusted cumulative incidence[a]		Absolute risk difference	
	Discontinued[b]	Not discontinued	Discontinued [vs. not discontinued]	Discontinued [vs. not discontinued]
Without opioid exposure[c]				
18-64 years	1.7 (1.6, 1.9)	1.3 (1.2, 1.4)	0.5 (0.3, 0.6)	1.4 (1.3, 1.5)
≥65 years	9.4 (9.2, 9.8)	5.9 (5.8, 6.1)	3.5 (3.3, 3.9)	1.6 (1.5, 1.7)
With opioid exposure				
18-64 years	3.2 (2.9, 3.5)	2.2 (2.0, 2.4)	1.0 (0.8, 1.3)	1.5 (1.3, 1.6)
≥65 years	10.5 (10.0, 11.1)	6.5 (6.2, 6.8)	4.1 (3.5, 4.5)	1.6 (1.5, 1.7)

[a] Outcome measure (mortality) computed at the end of follow-up (i.e., 360 days)
[b] Discontinuation defined as 31 consecutive days without prescription benzodiazepine coverage
[c] Opioid exposure defined as presence of at least one prescription opioid filled during the last 30 days of the baseline period.

© 2023 Maust DT et al. *JAMA Network Open.*

Table e8. Characteristics of Patients Prescribed Stable Long-term Benzodiazepine Therapy Stratified by Opioid Exposure at the End of the Grace Period for the **Per-Protocol Analysis**, Overall and by Benzodiazepine Discontinuation Status[a]

	**Opioid Exposure[b]**					
	**No**			**Yes**		
**Characteristic, N (%) or mean (SD)**	**Overall**	**Discontinued**	**Not Discontinued**	**Overall**	**Discontinued**	**Not Discontinued**
N (unweighted)	159848 (100)	18924 (11.8)	140924 (88.2)	108269 (100)	11637 (10.7)	96632 (89.3)
Age						
18-44	18496 (11.6)	2428 (12.8)	16068 (11.4)	10620 (9.8)	1233 (10.6)	9387 (9.7)
45-64	60329 (37.7)	6463 (34.2)	53866 (38.2)	51710 (47.8)	5244 (45.1)	46466 (48.1)
65+	81023 (50.7)	10033 (53.0)	70990 (50.4)	45939 (42.4)	5160 (44.3)	40779 (42.2)
Female	103112 (64.5)	11933 (63.1)	91179 (64.7)	70956 (65.5)	7388 (63.5)	63568 (65.8)
Race/ethnicity						
Asian	2196 (1.4)	291 (1.5)	1905 (1.4)	993 (0.9)	116 (1.0)	877 (0.9)
Black	14407 (9.0)	1651 (8.7)	12756 (9.1)	12354 (11.4)	1387 (11.9)	10967 (11.3)
Hispanic	17342 (10.8)	2070 (10.9)	15272 (10.8)	9373 (8.7)	1051 (9.0)	8322 (8.6)
White	125903 (78.8)	14912 (78.8)	110991 (78.8)	85549 (79.0)	9083 (78.1)	76466 (79.1)
Region						
Midwest	32968 (20.6)	3471 (18.3)	29497 (20.9)	20782 (19.2)	1908 (16.4)	18874 (19.5)
Northeast	15675 (9.8)	1553 (8.2)	14122 (10.0)	7880 (7.3)	676 (5.8)	7204 (7.5)
South	81663 (51.1)	9297 (49.1)	72366 (51.4)	59744 (55.2)	6403 (55.0)	53341 (55.2)
West	29542 (18.5)	4603 (24.3)	24939 (17.7)	19863 (18.3)	2650 (22.8)	17213 (17.8)
Anxiety	82237 (51.4)	9069 (47.9)	73168 (51.9)	61611 (56.9)	6263 (53.8)	55348 (57.3)
Depression	44853 (28.1)	5172 (27.3)	39681 (28.2)	33919 (31.3)	3632 (31.2)	30287 (31.3)
Chronic pain	114529 (71.6)	13952 (73.7)	100577 (71.4)	97498 (90.1)	10742 (92.3)	86756 (89.8)
Insomnia	26501 (16.6)	3645 (19.3)	22856 (16.2)	19749 (18.2)	2505 (21.5)	17244 (17.8)
Bipolar	13337 (8.3)	1476 (7.8)	11861 (8.4)	9627 (8.9)	920 (7.9)	8707 (9.0)
Other psychotic disorders	6227 (3.9)	874 (4.6)	5353 (3.8)	3622 (3.3)	429 (3.7)	3193 (3.3)
Substance use disorders						
Alcohol	4024 (2.5)	641 (3.4)	3383 (2.4)	3086 (2.9)	381 (3.3)	2705 (2.8)
Opioid	4204 (2.6)	826 (4.4)	3378 (2.4)	9141 (8.4)	1294 (11.1)	7847 (8.1)
Stimulant	691 (0.4)	150 (0.8)	541 (0.4)	684 (0.6)	108 (0.9)	576 (0.6)
Sedative	4120 (2.6)	578 (3.1)	3542 (2.5)	2667 (2.5)	343 (2.9)	2324 (2.4)
Cannabis	1006 (0.6)	188 (1.0)	818 (0.6)	922 (0.9)	142 (1.2)	780 (0.8)
Other substances	2098 (1.3)	409 (2.2)	1689 (1.2)	3157 (2.9)	458 (3.9)	2699 (2.8)
Modified Elixhauser score[c]	2.65 (2.39)	2.79 (2.52)	2.63 (2.37)	3.15 (2.55)	3.33 (2.68)	3.13 (2.53)
Average BZD lor-eq mg/day	2.78 (2.49)	2.42 (2.37)	2.83 (2.50)	3.51 (2.89)	2.99 (2.70)	3.57 (2.90)
30-day medication prescriptions						
Antidepressants	8.02 (8.82)	7.51 (8.43)	8.09 (8.87)	8.75 (9.21)	8.28 (8.96)	8.80 (9.24)
Antiepileptics	2.73 (5.67)	2.72 (5.54)	2.73 (5.68)	3.88 (6.35)	4.06 (6.33)	3.86 (6.36)
Antipsychotics	1.75 (5.04)	1.58 (4.73)	1.77 (5.08)	1.63 (4.65)	1.41 (4.29)	1.65 (4.69)
Z-drugs	0.86 (2.95)	0.78 (2.76)	0.87 (2.97)	1.19 (3.42)	1.09 (3.24)	1.20 (3.44)
Year (at start of follow-up)						
2014	24259 (15.2)	3527 (18.6)	20732 (14.7)	18351 (16.9)	2141 (18.4)	16210 (16.8)
2015	24051 (15.0)	3428 (18.1)	20623 (14.6)	17170 (15.9)	1776 (15.3)	15394 (15.9)
2016	26220 (16.4)	3397 (18.0)	22823 (16.2)	19062 (17.6)	1972 (16.9)	17090 (17.7)
2017	33916 (21.2)	3725 (19.7)	30191 (21.4)	25558 (23.6)	2655 (22.8)	22903 (23.7)
2018	51402 (32.2)	4847 (25.6)	46555 (33.0)	28128 (26.0)	3093 (26.6)	25035 (25.9)

SD: standard deviation; BZD: benzodiazepine; lor-eq: lorazepam-equivalent

[a] Discontinuation is defined as 31 consecutive days without prescription benzodiazepine coverage. Characteristics determined at end of the grace period, by which time each patient has a maximum of one clone remaining.

[b] Opioid exposure is defined as the presence of at least one prescription opioid fill during the last 30 days of the baseline period.

[c] The Elixhauser score is modified to exclude depression, substance abuse, alcohol abuse, and psychosis as these are included as separate covariates.

© 2023 Maust DT et al. *JAMA Network Open.*

Table e9. Characteristics of Patients Prescribed Stable Long-term Benzodiazepine Therapy **without** Opioid Exposure[a] at the End of the Grace Period, Overall and by Discontinuation Status[b]: Before and After Weighting in the **Per-Protocol** Analysis

	**Unweighted**			**Weighted**		
**Characteristic, N (%) or mean (SD)**	**Overall**	**Discontinued**	**Not Discontinued**	**Overall**	**Discontinued**	**Not Discontinued**
**N**	159848 (100)	18924 (11.8)	140924 (88.2)	342669 (100)	135093 (39.4)	207578 (60.6)
Age						
18-44	18496 (11.6)	2428 (12.8)	16068 (11.4)	45499 (13.3)	17859 (13.2)	27641 (13.3)
45-64	60329 (37.7)	6463 (34.2)	53866 (38.2)	132080 (38.5)	49821 (36.9)	82260 (39.6)
65+	81023 (50.7)	10033 (53.0)	70990 (50.4)	165090 (48.2)	67413 (49.9)	97677 (47.1)
Female	103112 (64.5)	11933 (63.1)	91179 (64.7)	218640 (63.8)	85602 (63.4)	133038 (64.1)
Race/ethnicity						
Asian	2196 (1.4)	291 (1.5)	1905 (1.4)	4733 (1.4)	1861 (1.4)	2871 (1.4)
Black	14407 (9.0)	1651 (8.7)	12756 (9.1)	30593 (8.9)	12156 (9.0)	18438 (8.9)
Hispanic	17342 (10.8)	2070 (10.9)	15272 (10.8)	36843 (10.8)	14620 (10.8)	22223 (10.7)
White	125903 (78.8)	14912 (78.8)	110991 (78.8)	270501 (78.9)	106456 (78.8)	164045 (79.0)
Region						
Midwest	32968 (20.6)	3471 (18.3)	29497 (20.9)	70117 (20.5)	26263 (19.4)	43854 (21.1)
Northeast	15675 (9.8)	1553 (8.2)	14122 (10.0)	32880 (9.6)	12319 (9.1)	20561 (9.9)
South	81663 (51.1)	9297 (49.1)	72366 (51.4)	174549 (50.9)	68446 (50.7)	106103 (51.1)
West	29542 (18.5)	4603 (24.3)	24939 (17.7)	65124 (19.0)	28064 (20.8)	37060 (17.9)
Anxiety	82237 (51.4)	9069 (47.9)	73168 (51.9)	175457 (51.2)	67410 (49.9)	108046 (52.1)
Depression	44853 (28.1)	5172 (27.3)	39681 (28.2)	94664 (27.6)	36718 (27.2)	57945 (27.9)
Chronic pain	114529 (71.6)	13952 (73.7)	100577 (71.4)	245856 (71.7)	98170 (72.7)	147687 (71.1)
Insomnia	26501 (16.6)	3645 (19.3)	22856 (16.2)	58842 (17.2)	24038 (17.8)	34804 (16.8)
Bipolar	13337 (8.3)	1476 (7.8)	11861 (8.4)	28418 (8.3)	10986 (8.1)	17432 (8.4)
Other psychotic disorders	6227 (3.9)	874 (4.6)	5353 (3.8)	13570 (4.0)	5667 (4.2)	7903 (3.8)
Substance use disorders						
Alcohol	4024 (2.5)	641 (3.4)	3383 (2.4)	9467 (2.8)	4032 (3.0)	5435 (2.6)
Opioid	4204 (2.6)	826 (4.4)	3378 (2.4)	10082 (2.9)	4501 (3.3)	5581 (2.7)
Stimulant	691 (0.4)	150 (0.8)	541 (0.4)	1708 (0.5)	763 (0.6)	945 (0.5)
Sedative	4120 (2.6)	578 (3.1)	3542 (2.5)	8776 (2.6)	3732 (2.8)	5044 (2.4)
Cannabis	1006 (0.6)	188 (1.0)	818 (0.6)	2474 (0.7)	1084 (0.8)	1389 (0.7)
Other substances	2098 (1.3)	409 (2.2)	1689 (1.2)	5235 (1.5)	2321 (1.7)	2913 (1.4)
Modified Elixhauser score[c]	2.65 (2.39)	2.79 (2.52)	2.63 (2.37)	2.61 (3.52)	2.65 (6.58)	2.58 (2.87)
Average BZD lor-eq mg/day	2.78 (2.49)	2.42 (2.37)	2.83 (2.50)	2.73 (3.67)	2.61 (6.86)	2.80 (2.99)
30-day medication prescriptions						
Antidepressants	8.02 (8.82)	7.51 (8.43)	8.09 (8.87)	7.87 (12.71)	7.69 (22.70)	7.99 (10.68)
Antiepileptics	2.73 (5.67)	2.72 (5.54)	2.73 (5.68)	2.70 (8.19)	2.72 (14.80)	2.69 (6.83)
Antipsychotics	1.75 (5.04)	1.58 (4.73)	1.77 (5.08)	1.70 (7.24)	1.66 (12.99)	1.72 (6.06)
Z-drugs	0.86 (2.95)	0.78 (2.76)	0.87 (2.97)	0.88 (4.36)	0.83 (7.69)	0.91 (3.68)
Year (at start of follow-up)						
2014	24259 (15.2)	3527 (18.6)	20732 (14.7)	58262 (17.0)	23667 (17.5)	34594 (16.7)
2015	24051 (15.0)	3428 (18.1)	20623 (14.6)	53874 (15.7)	22269 (16.5)	31605 (15.2)
2016	26220 (16.4)	3397 (18.0)	22823 (16.2)	57889 (16.9)	23100 (17.1)	34790 (16.8)
2017	33916 (21.2)	3725 (19.7)	30191 (21.4)	71243 (20.8)	27588 (20.4)	43655 (21.0)
2018	51402 (32.2)	4847 (25.6)	46555 (33.0)	101402 (29.6)	38468 (28.5)	62934 (30.3)

SD: standard deviation; BZD: benzodiazepine; lor-eq: lorazepam-equivalent
[a] Opioid exposure is defined as the presence of at least one prescription opioid fill during the last 30 days of the baseline period.
[b] Discontinuation is defined as 31 consecutive days without prescription benzodiazepine coverage. Characteristics determined at end of the grace period, by which time each patient has a maximum of one clone remaining.
[c] The Elixhauser score is modified to exclude depression, substance abuse, alcohol abuse, and psychosis as these are included as separate covariates.

© 2023 Maust DT et al. *JAMA Network Open.*

Table e10. Characteristics of Patients Prescribed Stable Long-term Benzodiazepine Therapy **with** Opioid Exposure[a] at the End of the Grace Period, Overall and by Discontinuation Status[b]: Before and After Weighting in the **Per-Protocol** Analysis

	**Unweighted**			**Weighted**		
**Characteristic, N (%) or mean (SD)**	**Overall**	**Discontinued**	**Not Discontinued**	**Overall**	**Discontinued**	**Not Discontinued**
**N**	108269 (100)	11637 (10.7)	96632 (89.3)	225007 (100)	88070 (39.1)	136938 (60.9)
Age						
18-44	10620 (9.8)	1233 (10.6)	9387 (9.7)	24400 (10.8)	9304 (10.6)	15096 (11.0)
45-64	51710 (47.8)	5244 (45.1)	46466 (48.1)	108491 (48.2)	41390 (47.0)	67102 (49.0)
65+	45939 (42.4)	5160 (44.3)	40779 (42.2)	92116 (40.9)	37376 (42.4)	54740 (40.0)
Female	70956 (65.5)	7388 (63.5)	63568 (65.8)	145544 (64.7)	56015 (63.6)	89530 (65.4)
Race/ethnicity						
Asian	993 (0.9)	116 (1.0)	877 (0.9)	2030 (0.9)	765 (0.9)	1265 (0.9)
Black	12354 (11.4)	1387 (11.9)	10967 (11.3)	25699 (11.4)	10236 (11.6)	15463 (11.3)
Hispanic	9373 (8.7)	1051 (9.0)	8322 (8.6)	19683 (8.7)	7920 (9.0)	11763 (8.6)
White	85549 (79.0)	9083 (78.1)	76466 (79.1)	177595 (78.9)	69148 (78.5)	108447 (79.2)
Region						
Midwest	20782 (19.2)	1908 (16.4)	18874 (19.5)	42458 (18.9)	15394 (17.5)	27063 (19.8)
Northeast	7880 (7.3)	676 (5.8)	7204 (7.5)	15734 (7.0)	5627 (6.4)	10107 (7.4)
South	59744 (55.2)	6403 (55.0)	53341 (55.2)	124631 (55.4)	49173 (55.8)	75457 (55.1)
West	19863 (18.3)	2650 (22.8)	17213 (17.8)	42185 (18.7)	17875 (20.3)	24310 (17.8)
Anxiety	61611 (56.9)	6263 (53.8)	55348 (57.3)	127263 (56.6)	48807 (55.4)	78456 (57.3)
Depression	33919 (31.3)	3632 (31.2)	30287 (31.3)	69724 (31.0)	27213 (30.9)	42511 (31.0)
Chronic pain	97498 (90.1)	10742 (92.3)	86756 (89.8)	204080 (90.7)	80503 (91.4)	123577 (90.2)
Insomnia	19749 (18.2)	2505 (21.5)	17244 (17.8)	42718 (19.0)	17332 (19.7)	25386 (18.5)
Bipolar	9627 (8.9)	920 (7.9)	8707 (9.0)	19461 (8.6)	7274 (8.3)	12186 (8.9)
Other psychotic disorders	3622 (3.3)	429 (3.7)	3193 (3.3)	7528 (3.3)	2984 (3.4)	4543 (3.3)
Substance use disorders						
Alcohol	3086 (2.9)	381 (3.3)	2705 (2.8)	6762 (3.0)	2778 (3.2)	3984 (2.9)
Opioid	9141 (8.4)	1294 (11.1)	7847 (8.1)	20043 (8.9)	8234 (9.3)	11809 (8.6)
Stimulant	684 (0.6)	108 (0.9)	576 (0.6)	1601 (0.7)	664 (0.8)	937 (0.7)
Sedative	2667 (2.5)	343 (2.9)	2324 (2.4)	5821 (2.6)	2523 (2.9)	3298 (2.4)
Cannabis	922 (0.9)	142 (1.2)	780 (0.8)	2086 (0.9)	908 (1.0)	1179 (0.9)
Other substances	3157 (2.9)	458 (3.9)	2699 (2.8)	7212 (3.2)	3001 (3.4)	4211 (3.1)
Modified Elixhauser score[c]	3.15 (2.55)	3.33 (2.68)	3.13 (2.53)	3.13 (3.69)	3.17 (7.14)	3.11 (3.02)
Average BZD lor-eq mg/day	3.51 (2.89)	2.99 (2.70)	3.57 (2.90)	3.40 (4.13)	3.22 (7.90)	3.52 (3.40)
30-day medication prescriptions						
Antidepressants	8.75 (9.21)	8.28 (8.96)	8.80 (9.24)	8.55 (13.11)	8.33 (24.67)	8.69 (10.92)
Antiepileptics	3.88 (6.35)	4.06 (6.33)	3.86 (6.36)	3.90 (9.10)	3.96 (17.16)	3.86 (7.56)
Antipsychotics	1.63 (4.65)	1.41 (4.29)	1.65 (4.69)	1.56 (6.52)	1.49 (12.13)	1.60 (5.47)
Z-drugs	1.19 (3.42)	1.09 (3.24)	1.20 (3.44)	1.20 (4.95)	1.15 (9.19)	1.24 (4.16)
Year (at start of follow-up)						
2014	18351 (16.9)	2141 (18.4)	16210 (16.8)	40923 (18.2)	15758 (17.9)	25165 (18.4)
2015	17170 (15.9)	1776 (15.3)	15394 (15.9)	35435 (15.7)	13692 (15.5)	21743 (15.9)
2016	19062 (17.6)	1972 (16.9)	17090 (17.7)	39230 (17.4)	14956 (17.0)	24275 (17.7)
2017	25558 (23.6)	2655 (22.8)	22903 (23.7)	52464 (23.3)	20730 (23.5)	31734 (23.2)
2018	28128 (26.0)	3093 (26.6)	25035 (25.9)	56955 (25.3)	22933 (26.0)	34022 (24.8)

SD: standard deviation; BZD: benzodiazepine; lor-eq: lorazepam-equivalent

[a] Opioid exposure is defined as the presence of at least one prescription opioid fill during the last 30 days of the baseline period.

[b] Discontinuation is defined as 31 consecutive days without prescription benzodiazepine coverage. Characteristics determined at end of the grace period, by which time each patient has a maximum of one clone remaining.

[c] The Elixhauser score is modified to exclude depression, substance abuse, alcohol abuse, and psychosis as these are included as separate covariates.

© 2023 Maust DT et al. *JAMA Network Open.*

Table e11. Adjusted Incidence, Risk Difference, and Risk Ratio of Mortality and Secondary Outcomes Among Patients Prescribed Stable Long-term Benzodiazepine Therapy, Stratified by Opioid Exposure, By Treatment Strategy

	%			Risk ratio
	**Adjusted cumulative incidence[a]**		**Absolute risk difference**	
	**Discontinued[b]**	**Not discontinued**	**Discontinued [vs. not discontinued]**	**Discontinued [vs. not discontinued]**
**All-Cause Mortality**				
Without opioid exposure[c]				
Intention-to-treat	5.5 (5.4, 5.8)	3.5 (3.4, 3.6)	2.1 (1.9, 2.3)	1.6 (1.6, 1.7)
Per-protocol	7.5 (7.3, 7.9)	3.3 (3.2, 3.4)	4.3 (4.0, 4.6)	2.3 (2.2, 2.4)
With opioid exposure				
Intention-to-treat	6.3 (6.0, 6.6)	3.9 (3.8, 4.1)	2.4 (2.2, 2.7)	1.6 (1.5, 1.7)
Per-protocol	7.9 (7.4, 8.4)	3.7 (3.6, 3.9)	4.1 (3.7, 4.6)	2.1 (2.0, 2.2)
**Secondary Outcomes**				
**Non-Fatal Overdose**				
Without opioid exposure				
Intention-to-treat	1.1 (1.0, 1.1)	0.9 (0.9, 1.0)	0.1 (0.1,0.2)	1.2 (1.1, 1.3)
Per-protocol	1.2 (1.1, 1.3)	0.9 (0.9, 1.0)	0.3 (0.2, 0.4)	1.3 (1.2, 1.4)
With opioid exposure				
Intention-to-treat	2.2 (2.0, 2.3)	1.8 (1.7, 1.9)	0.4 (0.2, 0.5)	1.2 (1.1, 1.3)
Per-protocol	2.5 (2.3, 2.8)	1.8 (1.7, 1.9)	0.7 (0.5, 1.0)	1.4 (1.3, 1.5)
**Suicide Attempt or Self-Inflicted Injury**				
Without opioid exposure				
Intention-to-treat	0.5 (0.5, 0.6)	0.5 (0.4, 0.5)	0.1 (0.0, 0.1)	1.1 (1.0, 1.2)
Per-protocol	0.6 (0.5, 0.7)	0.5 (0.4, 0.5)	0.1 (0.0, 0.2)	1.3 (1.1, 1.5)
With opioid exposure				
Intention-to-treat	0.8 (0.7, 0.9)	0.7 (0.6, 0.7)	0.1 (0.0, 0.2)	1.2 (1.1, 1.4)
Per-protocol	0.9 (0.8, 1.1)	0.7 (0.6, 0.7)	0.3 (0.1, 0.4)	1.4 (1.2, 1.6)
**Suicidal Ideation**				
Without opioid exposure				
Intention-to-treat	1.0 (0.9, 1.1)	0.8 (0.7, 0.8)	0.3 (0.2, 0.3)	1.4 (1.2, 1.5)
Per-protocol	1.2 (1.1, 1.4)	0.7 (0.7, 0.8)	0.5 (0.3, 0.6)	1.6 (1.4, 1.9)
With opioid exposure				
Intention-to-treat	1.3 (1.1, 1.4)	0.9 (0.9, 1.0)	0.4 (0.2, 0.5)	1.4 (1.2, 1.5)
Per-protocol	1.5 (1.3, 1.7)	0.9 (0.8, 1.0)	0.6 (0.4, 0.8)	1.7 (1.5, 1.9)
**Emergency Department Use**				
Without opioid exposure				
Intention-to-treat	42.7 (42.4, 43.1)	36.6 (36.4, 36.9)	6.1 (5.7, 6.5)	1.2 (1.2, 1.2)
Per-protocol	48.1 (47.4, 48.7)	36.8 (36.6, 37.1)	11.2 (10.6, 11.8)	1.3 (1.3, 1.3)
With opioid exposure				
Intention-to-treat	54.3 (53.8, 54.8)	45.2 (44.9, 45.5)	9.1 (8.5, 9.5)	1.2 (1.2, 1.2)
Per-protocol	60.2 (59.4, 61.0)	45.3 (45.1, 45.7)	14.9 (14.1, 15.6)	1.3 (1.3, 1.3)

CI, confidence interval
[a] Outcome measure (i.e., mortality) computed at the end of follow-up (i.e., 360 days)
[b] Discontinuation is defined as 31 consecutive days without prescription benzodiazepine coverage
[c] Opioid exposure defined as presence of at least one prescription opioid filled during the last 30 days of the baseline period.

© 2023 Maust DT et al. *JAMA Network Open.*

Table e12. Results of Sensitivity Analysis Examining Benzodiazepine Discontinuation and Mortality Risk Among Patients Prescribed Stable Long-term Benzodiazepine Therapy, Stratified by Opioid Exposure

	%			Risk ratio
	Adjusted cumulative incidence[a]		Absolute risk difference	
	Discontinued[b]	Not discontinued	Discontinued [vs. not discontinued]	Discontinued [vs. not discontinued]
Without opioid exposure[c]				
Intention-to-treat	6.8 (6.6, 7.0)	3.6 (3.5, 3.7)	3.2 (3.0, 3.4)	1.9 (1.8, 1.9)
Per-protocol	8.0 (7.7, 8.4)	3.5 (3.4, 3.6)	4.5 (4.2, 4.8)	2.3 (2.2, 2.4)
With opioid exposure				
Intention-to-treat	7.8 (7.3, 8.2)	4.1 (4.0, 4.2)	3.7 (3.3, 4.1)	1.9 (1.8, 2.0)
Per-protocol	8.7 (8.1, 9.2)	4.0 (3.8, 4.1)	4.7 (4.2, 5.2)	2.2 (2.1, 2.3)

[a] Outcome measure (mortality) computed at the end of follow-up (i.e., 360 days)
[b] Discontinuation defined as 61 consecutive days without prescription benzodiazepine coverage
[c] Opioid exposure defined as presence of at least one prescription opioid filled during the last 30 days of the baseline period

© 2023 Maust DT et al. *JAMA Network Open.*
