# Supplementary material for: Benzodiazepine Discontinuation and Mortality Among Patients Receiving Long-Term Benzodiazepine Therapy
Source: JAMA Netw Open. 2023 Dec 20;6(12):e2348557. doi: 10.1001/jamanetworkopen.2023.48557 (PMC10733804; doi:10.1001/jamanetworkopen.2023.48557)
Supplement: Supplement 2. — Data Sharing Statement [file jamanetwopen-e2348557-s002.pdf]

Data Sharing Statement

Maust. Benzodiazepine Discontinuation and Mortality Among Patients on Long-Term Benzodiazepine Therapy. *JAMA Netw Open*. Published December 20, 2023. doi:10.1001/jamanetworkopen.2023.48557

Data
Data available: No

Additional Information
Explanation for why data not available: Under the teams of the University's data use agreement, these data may not be shared.
